# Giant magnetic moment increase by ultrafast laser light

**DOI:** 10.1038/s41467-026-73780-z

**Published:** 2026-06-03

**Authors:** Sangeeta Sharma, Deepika Gill, Jyoti Krishna, Eddie Harris-Lee, John Kay Dewhurst, Sam Shallcross

**Affiliations:** 1 Max-Born-Institut für Nichtlineare Optik und Kurzzeitspektroskopie, Berlin, Germany; 2 Institute for theoretical solid-state physics and Halle-Berlin-Regensburg Cluster of Excellence CCE, Freie Universität Berlin, Berlin, Germany; 3 Max-Planck-Institut fur Mikrostrukturphysik Weinberg 2, Halle, Germany

**Keywords:** Ferromagnetism, Nonlinear optics

## Abstract

It is now well established that a few femtosecond laser pulse will induce an ultrafast loss of moment in a magnetic material. Here we show that the opposite effect can also occur: an ultrafast increase in moment. Employing both tight-binding and state-of-the-art time dependent density functional theory we find that laser light tuned to the majority spin conduction band in the 2d magnets CrI_3_ and CrSBr generates an ultrafast giant moment increase, of up to 33% in the case of CrI_3_ (2 *μ*_*B*_). Underpinning this is spin-orbit induced valence band spin texture that, in combination with a strong field light pulse, facilitates an optical spin flip transition involving both intra- and inter-band excitation. Our findings, that establish a general mechanism by which ultrafast light pulses may enhance as well as decrease the magnetic moment, point towards rich possibilities for light control over magnetic matter at femtosecond times.

## Abstract (web summary)

It is well established that intense laser pulses can demagnetize magnetic matter; however, the inverse process of magnetic moment enhancement by ultrafast laser light is generally precluded. Here, Sharma and coauthors show how combining a spin-orbit-induced spin texture with strong optical drive in CrSBr and CrI_3_ leads to substantial increase in magnetic moment.

## Introduction

It is almost paradigmatic that the moments of transition metal magnets and their compounds will decrease upon irradiation by an ultrafast pulse of intense laser light ^1–6^. While longer (picosecond to nanosecond) time scales can unveil a contrary picture, for example a light induced increase in Curie temperature^7,8^, such effects are driven by lightwave coupling to the lattice degrees of freedom or current flow between components of a magnetic heterostructure. At the attosecond to few femtosecond times dominated by purely electronic interactions, such physics is precluded. Control over the strength of magnetic order by light thus appears—for the ultrafast few femtosecond regime in which interesting coherent quantum effects may occur—to stand comparatively impoverished.

Here we demonstrate that, surprisingly, for the two dimensional (2d) magnets CrI_3_^9–11^ and CrBrS^12–14^ this is not true: a giant and ultrafast increase in magnetic moment can be induced by a laser pulse, with an enhancement of up to 2*μ*_*B*_ found in CrI_3_. Underpinning this effect is the fact that strong spin-orbit coupling imbues the valence band manifolds of these materials with a spin texture, in which the direction of the moment can evolve from up to down within a single band^11^. A light pulse generating *intra*-band evolution of crystal momentum can thus induce *intra*-band spin rotation within the valence band. This forms the basis of an optical excitation—which as a convenient shorthand we denote an “intraflip"—combining an intra-band rotation and inter-band excitation that flips the spin while, just as in a direct optical transition, preserving the crystal momentum.

We show that such intraflips can drive a giant moment increase at time scales from the few femtosecond single cycle limit to much longer multi-cycle pulses of  ~100 fs duration, with both linearly and circularly polarized light generating moment increase; the effect should therefore be observable employing present day standard ultrafast laser pulses. Ab-initio calculation of the transient XMCD from the Cr L_2,3_ edge reveals clear signatures of this moment increase, allowing experimental probing of our predictions. Requiring only (i) spin-orbit induced spin texture in the valence band and (ii) a pure majority spin conduction band the effect we propose is general, and will apply to any material that fulfils these conditions. Our work thus both highlights the control possibilities of light wave coupling to a non-trivial spin texture, as well as revealing an unexpected richness of magnetic control possible in the ultrafast regime of laser induced spin dynamics.

## Results

We first consider the two dimensional semi-conductor CrI_3_^9,10^, whose ferromagnetic order (Curie temperature 45 K) has revealed a rich physics of low dimensional magnetism with remarkable light-matter interaction^15–18^ and a rich coupling between magnetic, structural, and electronic order, both in a moiré geometry^19–23^ and in single layer systems^24,25^. While this low Curie temperature may preclude long time measurement of light induced changes in magnetism, as orientational disorder will set in at picosecond times, the robust local moment permits observation of ultrafast spin dynamics via XMCD or MOKE transient spectroscopy. Indeed, several recent experimental works have demonstrating light induced spin switching in CrI_3_^26–28^, demonstrating that this material represents a practical experimental platform for investigating ultrafast spin dynamics. Subsequently, we will demonstrate that similar physics is found in the recently discovered 2d magnet CrBrS^12–14^, that possesses a significantly higher Curie temperature of 132 K.

To explore the ultrafast spin dynamics of these two materials we employ a Wannierized tight-binding scheme, with key results verified by ab-initio full potential time-dependent density functional theory. Computational details of both these approaches are presented in the Supplemental document.

**Fig. 1:**
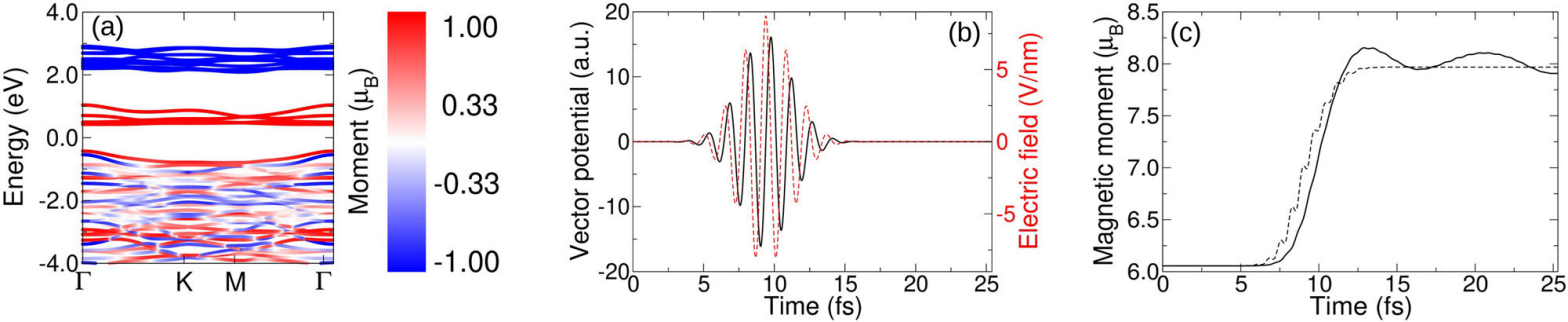
Ultrafast light induced increase of magnetic moment in CrI_3_. **a** The band structure of CrI_3_ with color denoting the z-component of the spin moment (the other components are zero). **b** The vector potential of a 2.8 eV linearly polarized pulse, presented in atomic units. **c** Irradiation of CrI_3_ by this pulse generates an increase in magnetic moment from the ground state value of 6 *μ*_*B*_ to 8 *μ*_*B*_. Here, the full line denotes the expectation value of the spin operator (i.e. the full magnetic moment), with the broken line the intra-band contribution to this moment (see Supplemental section 7 for a discussion of these terms). The pulse fluence is 8.6 mJ/cm^2^ with peak electric field 8.7 V/nm.

The band structure of CrI_3_ is shown in Fig. 1a, with the band color indicating the spin moment (the Fermi energy is set to zero). Two distinctive features can be observed: (i) the conduction bands are either pure spin up or pure spin down while (ii) the valence bands exhibit a more complex spin structure, with continuous evolution of spin up to spin down character within the same band.

Excitation by a 2.8 eV linearly polarized laser pulse, full width half maximum (FWHM) 4.2 fs and fluence 8.6 mJ/cm^2^, vector potential shown in panel (b), yields, in dramatic contrast to the “expected" behavior of demagnetization, an increase in moment from 6.06 *μ*_*B*_/unit cell to  ~ 8 *μ*_*B*_, panel (c). It should be stressed that direct optical transitions, i.e. transitions from valence (VB) to conduction (CB) at the *same* k-vector involving matrix elements 〈*c*_**k**_∣*δH*∣*v*_**k**_〉, cannot generate a change of moment: such transitions are spin preserving as the light-matter coupling Hamiltonian *δH* does not involve spin. A quite different mechanism of charge excitation must therefore underpin the ultrafast giant moment increase seen here.

**Fig. 2:**
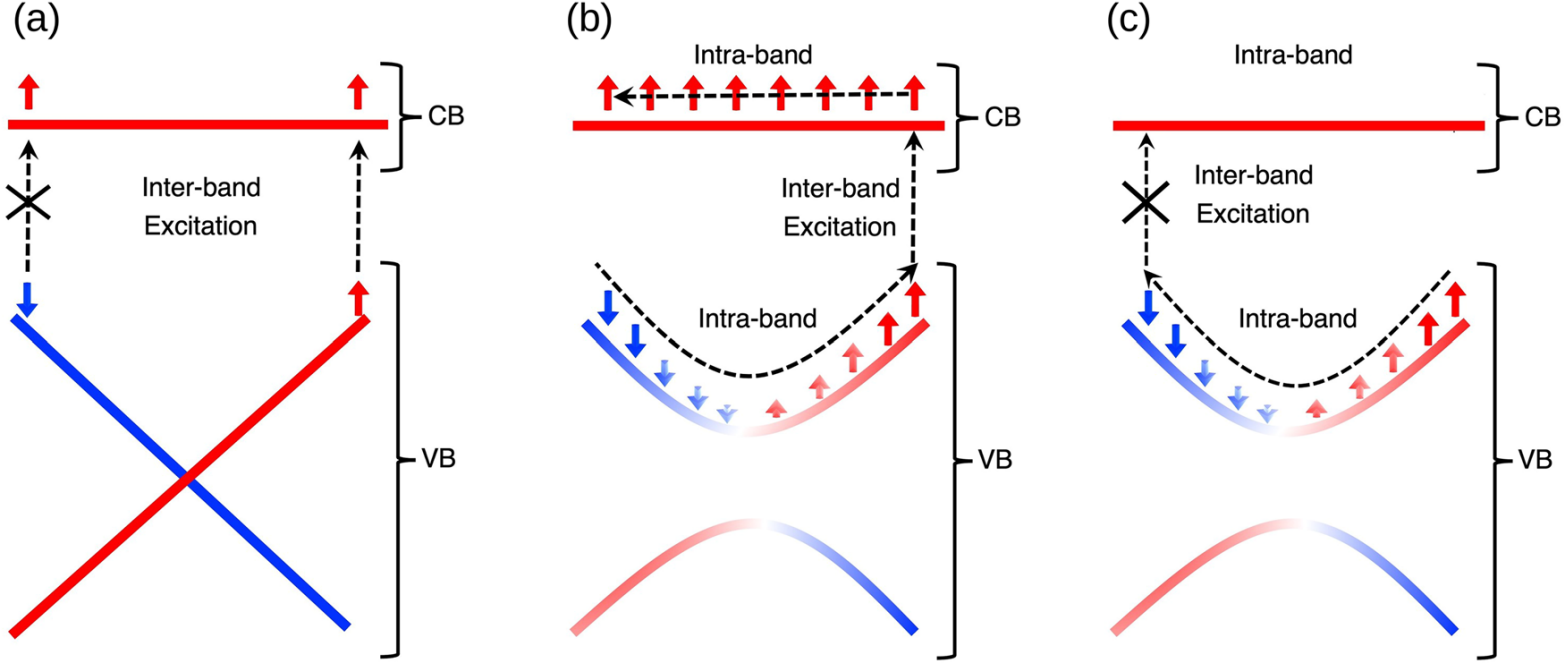
Intraflip mechanism underpinning light induced magnetic moment increase. In the absence of spin-orbit coupling optical excitation from spin down to spin up bands is forbidden, (**a**). However, if strong spin-orbit coupling imparts a spin texture to the spin hybridized valence bands (VB), (**b**), then an excitation process in which a spin down state is excited to spin up state is made possible, indicated by the broken line. In this three stage mechanism (i) light induced intra-band excitation evolves a spin state up to a spin down state *within* the valence band, followed by (ii) inter-band excitation to the conduction band (CB), allowed as both states are now spin up, with (iii) intra-band evolution then returning this state to its initial crystal momentum while retaining the spin up character as the CB is pure spin up. The overall result is thus a spin flip excitation that preserves the crystal momentum. **c** Inverting the dynamical pathway yields no excitation as this then involves a forbidden direct spin down to up transition.

This is provided for by the fact that strong light pulses generate intra-band evolution of crystal momentum, and not only direct transition from valence to conduction. This excitation mechanism is illustrated schematically in Fig. 2, where we consider the simplest possible representation of the CrI_3_ band structure: spin hybridized valence bands and a pure spin up conduction band. In the absence of spin-orbit coupling opposite spin bands are orthogonal, and excitation from the spin down valence band to the spin up conduction band forbidden, panel (a). Strong spin orbit coupling dramatically changes this: valence bands now evolve spin character continuously from up to down, allowing an optical excitation that can flip the spin, indicated in Fig. 2b by the broken line. This consists of a sequence of three excitation steps: (i) intra-band evolution of a spin down state to a spin up state within the valence band; (ii) inter-band excitation to the conduction band; and finally (iii) intra-band motion returning the state to its initial momentum while preserving the spin up state. The net result is an optical transition that, at a given crystal momentum **k**, excites a spin down valence state to a spin up conduction band state.

To date ultrafast (i.e. tens of femtoseconds) spin dynamics has been understood in terms of charge excitation driven by direct optical excitation, followed by spin-orbit induced spin flips. This two step scheme underpins present day understanding of ultrafast demagnetization^3,29,30^. The picture that emerges here is quite different: intraflip excitations, via non-perturbative intra-band evolution of momentum, exploit the spin-orbit induced spin structure of the ground state to generate a light induced increase in moment. The understanding of the distinct physics of intra- and inter-band transitions in ultra-fast laser excitation has been extensively explored in the context of high harmonic generation^31^, here both aspects combine to create a spin-flip mechanism in solids.

**Fig. 3:**
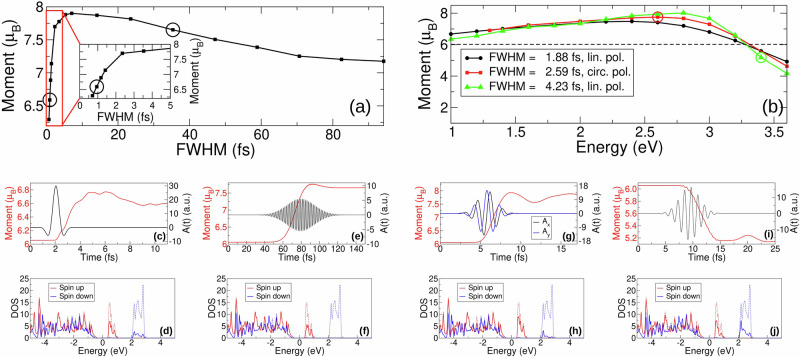
Ultrafast moment control in CrI_3_. **a** Variation of pulse duration while holding the fluence (5.84 mJ/cm^2^) and central frequency (2.4 eV) fixed reveals that light induced increase in moment occurs from sub-cycle attosecond times to long time multi-cycle pulse, with the inset figure a zoom of the few femtosecond regime. The pulse peak electric field ranges between 5.2 and 18.6 V/nm. **b** Variation of frequency unveils regimes of both increase and decrease of moment: below  ~ 3.2 eV the pulse generates an increase in moment, while for higher frequencies a reduction in moment is observed. Note that similar behavior is observed for both linearly and circularly polarized light pulses. **c** shows the vector potential and temporal evolution of the increasing magnetic moment for a single cycle pulse, with **d** the density of states (DOS) after the pulse. **e**,**f** present corresponding data for a multi-cycle pulse. Both (**c**,**d**) and (**e**,**f**) correspond to the open circles as indicated in (**a**). **g–j** present similar data for the cases of moment increase by circularly polarized light, and moment decrease by a higher frequency linearly polarized pulse; these cases correspond to the open circles shown in (**b**).

Having established a light induced ultrafast increase in moment we now explore (i) the temporal limits of this phenomena and (ii) how pulse parameters can be used to exert control over the moment dynamics. To this end we first vary the laser pulse duration while holding the frequency fixed at 2.4 eV. We further fix the fluence to a value of 5.84 mJ/cm^2^, thereby enforcing a “trade off" between pulse amplitude and duration; longer pulses, to preserve fluence, must have reduced amplitude. As it is the pulse vector potential amplitude determines the magnitude of the light induced intra-band momentum evolution, the intraflip mechanism outlined above would suggest that trading pulse amplitude for duration in this way would reduce the efficiency of the laser induced moment increase. This can be seen in Fig. 3a, in which a reduction in the moment enhancement occurs on increasing pulse duration: a moment enhancement of 7.90*μ*_*B*_ at 8 fs duration falls off to 7.18*μ*_*B*_ at 93 fs. At the opposite temporal extreme, the sub-cycle limit (a single cycle of 2.4 eV light corresponds to 1.73 fs) reveals a persisting moment enhancement of up to 1.0*μ*_*B*_ (in Section 5 of the Supplemental document we show that even attosecond pulses can achieve this moment increase). Remarkably, therefore, a robust light induced moment increase is found for over two orders of magnitude of pulse duration. Underlying the less efficient moment enhancement in the sub-cycle regime is the broadband range of frequencies contained in such short pulse envelopes, resulting in excitation also to the Cr *t*_2*g*_ minority states, Fig. 3c, d. In the long time limit, in contrast, excitation occurs only to the majority Cr *e*_*g*_ states, Fig. 3e, f.

Tuning of the pulse central frequency, Fig. 3b, reveals two distinct windows: below  ~ 3.2 eV a light induced increase of moment is observed, while above  ~ 3.2 eV a decrease in moment is found. This behavior is found for a broad range of pulse durations and structures, including both linearly and circularly polarized light. The occurrence of moment reduction arises as, at high pulse central frequency, intraflip excitations are detuned from the conduction band Cr majority and into conduction band Cr minority states, as may be seen from inspection of the post-pulse density of states, Fig. 3g–j. Note also that the post-pulse variation of the moment observed in Fig. 3 (and also in the ab-initio calculations to be described below), results not from an excitation process (the excited charge is constant post-pulse) but is driven by interference effects in the inter-band contribution to the dynamical magnetic moment, see Supplemental Section 7.

We now consider the light induced ultrafast increase in magnetic moment within the very different approach of time dependent density functional theory (TD-DFT). We employ the adiabatic local density approximation and treat the electronic structure within the full potential method as implemented within the Elk code; full numerical details are provided in the Supplemental document. In contrast to the tight-binding method in which the band structure and spin-orbit coupling remain fixed with dynamical evolution only via changing occupation numbers, full potential TD-DFT allows dynamics of both the effective Kohn-Sham potential—and thus the spin-orbit coupling—and the full density *ρ*(**r**, *t*). The method thus provides a rigorous test of results obtained via tight-binding calculations.

**Fig. 4:**
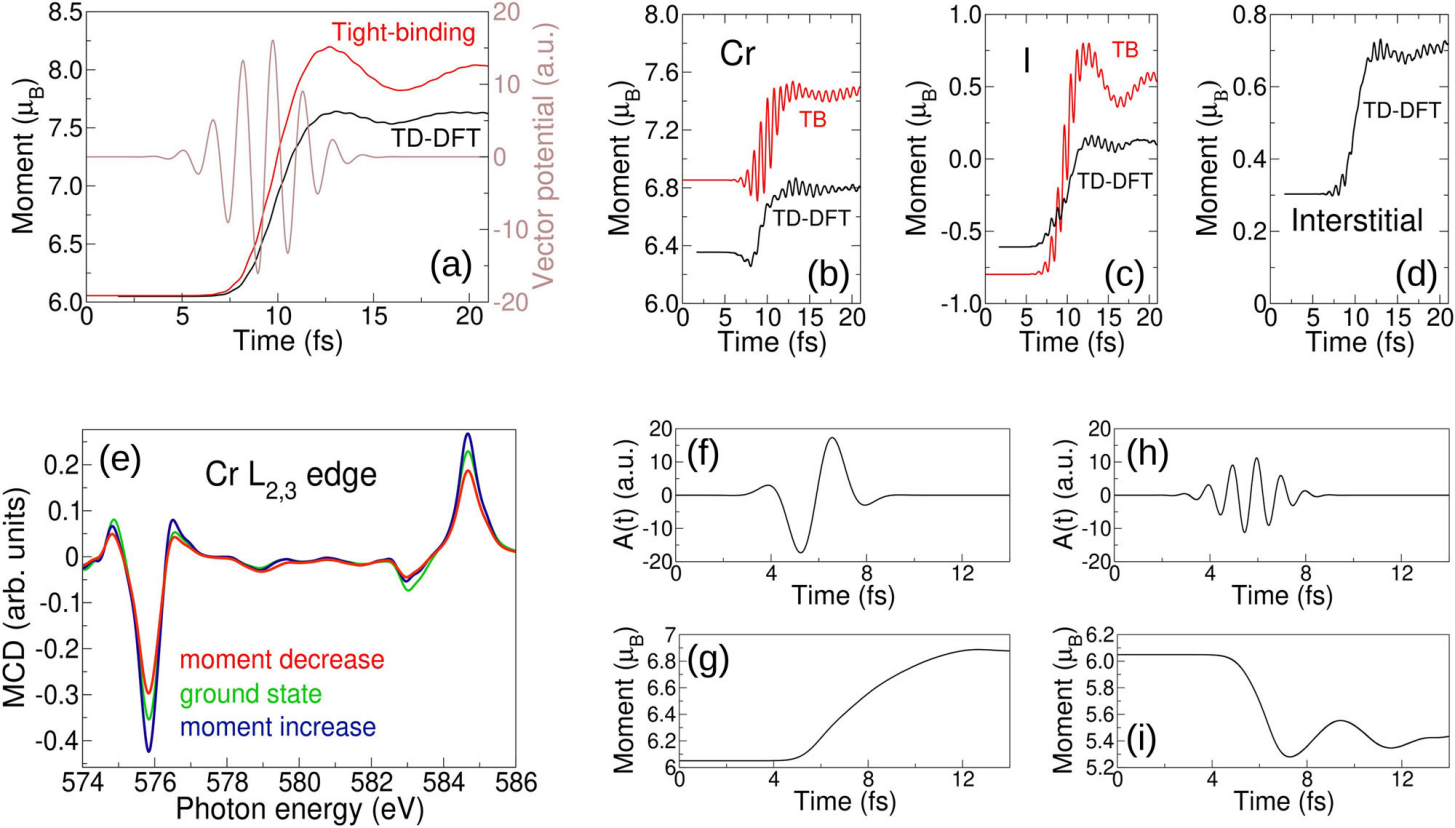
Time-dependent density functional theory (TD-DFT) calculations of ultrafast moment increase in CrI_3_. **a** The temporal dependence of the light induced moment calculated via TD-DFT and the tight-binding method, reveal a similar and substantial ultrafast increase of more than 1.5*μ*_*B*_. **b–d** The Cr, I, and interstitial components of the moment show qualitatively similar behavior, with the light pulse inducing a reversal of the I moment. The dichroic response of the Cr L_2,3_ edge, both in the ground state and after ultrafast light induced increase or decrease in moment, is presented in (**e**), with vector potential and transient moment shown in (**f–i**). The clear changes in the dichroic response demonstrate that the Cr L_2,3_ edge provides a sensitive experimental marker of the light induced increase or decrease in moment. The pulse parameters are: (**f**) a frequency of 1.3 eV, duration 2.6 fs, peak electric field 7.7 V/nm, and fluence 4.6 mJ/cm^2^; (**h**) a frequency of 4.0 eV, duration 2.6 fs, peak electric field 8.7 V/nm, and fluence 5.3 mJ/cm^2^.

In Fig. 4a, we present the transient moment for a 2.4 eV pulse (vector potential shown in gray) calculated both via the TD-DFT and tight-binding methods. A similar large ( > 1.5*μ*_*B*_) increase is seen, with the tight-binding method slightly over estimating the moment increase. A character analysis of the transient moment, Fig. 4b–d, reveals a qualitatively similar behavior in both methods, with a substantial component of the ultrafast moment increase resulting from spin reversal on the I atoms. Within TD-DFT the magnetization is treated as an unconstrained vector field, which can then be integrated around atomic centers to obtain the species resolved moments. The time dependence of the magnetic moment of the interstitial region, i.e., that part of space not included in the “muffin-tins" around each atomic site, is presented in Fig. 4d. The definition of species resolved moments is thus seen to be to some extent arbitrary, accounting for the excellent agreement between the Elk code (full potential basis) and the Quantum Espresso (pseudopotential basis) for the total moment, while differing on species projected moments.

As the TD-DFT calculations employ a full-potential method all states are included in the basis, and this allows for a determination of the x-ray magnetic circular dichroism (XMCD) at the Cr L-edge, which is  ~ 580 eV below the Fermi energy. Transient XMCD spectroscopy provides a sensitive tool of ultrafast changes in moment^32–37^, with TD-DFT shown to provide excellent agreement with experiment at early femtosecond times^33,38^. The measured ground state XMCD response^39^ can be employed to scissors correct the peak positions, allowing prediction of the transient XMCD as an experimental test. To this end we employ two circularly polarized pulses, that cover the two cases of light induced moment increase and light induced moment decrease, and calculate the XMCD signal after the pulse, Fig. 3e. This reveals distinctive changes of the XMCD response to the light induced increase or decrease in moment, thus providing an experimentally testable prediction of the novel moment increase in CrI_3_. The vector potentials and time dependent moment for the two cases of moment increase and decrease are shown in panels (f,g) and (h,i) respectively.

The present TD-DFT calculations do not include the dynamics of the lattice degrees of freedom, and thus the flow of spin angular momentum, via orbital degrees of freedom^40^, to the lattice^41^ is not described within our simulation. Nevertheless, the dynamics of spin and orbital angular momentum may provide insight both into the microscopic mechanism of ultrafast moment increase, and in particular how the gain in spin angular momentum is compensated for by orbital degrees of freedom. It should be pointed out that while the calculation of **L** in periodic solids at equilibrium is well established, in highly non-equilibrium situations a corresponding theory of changes in **L** does not at present exist.

**Fig. 5:**
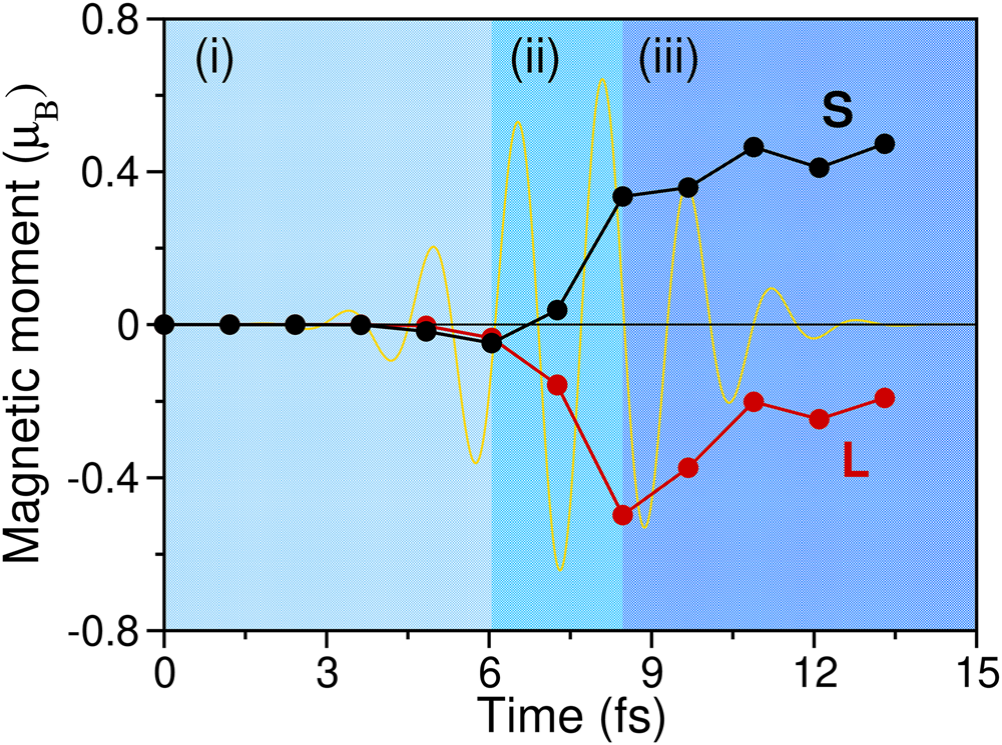
Early time spin and orbital angular momentum dynamics. Shown are the temporal change in spin **S** and orbital angular momentum **L** = **r** × **p**, both evaluated within the Cr muffin-tin, along with the laser pulse shown in background. Three regimes of spin and angular momentum dynamics can be discerned. (i) At early times both and exhibit a reduction, behavior similar to that seen in demagnetization dynamics of elemental Ni and Co. (ii) This, however, is succeeded by a regime in which the spin angular momentum increases - generated by intraflips associated with the intraband evolution of momentum - while the orbital angular momentum decreases. The microscopic muffin-tin currents associated with this reduction in **L** would, via forces acting on the nuclei, transfer this reduction in angular momentum to the lattice, thus ensuring conservation of angular momentum.

To bypass this we calculate **L**(*t*) and **S**(*t*) within the muffin-tins (MT) of each atomic species – a “poor mans" non-equilibrium **L**. This, shown in Fig. 5 for the Cr-MT, reveals three distinct temporal domains. (i) At early times before the peak of the pulse spin and angular momentum both begin very slightly to decrease, behavior that is reminiscent of the demagnetization dynamics of Ni and Co^40^. (ii) The “intraflip” mechanism then takes over, active at the peak of the pulse as this requires a large amplitude of the vector potential to drive intra-band evolution of crystal momentum, generating an increase in the spin angular momentum *S*. This increase in however is accompanied by a corresponding reduction in orbital angular momentum *L* (note that in Fig. 5 the change in these quantities is presented). The dynamical excitation thus has generated microscopic currents within the muffin-tin that to some extent compensate for the increase in *S*. These microscopic currents in turn generate forces on the nuclei which would then, if the nuclei were not clamped, induce nuclear dynamics transferring angular momentum from the orbital degrees of freedom of the electron system to the lattice. Thus ultimately the increase in *S* would be compensated for by a decrease in the angular momentum of the nuclear system. (iii) Finally we see that *L*(*t*) begins to increase, as these forces have acted on the clamped nuclei, while the spin continues to increase somewhat.

**Fig. 6:**
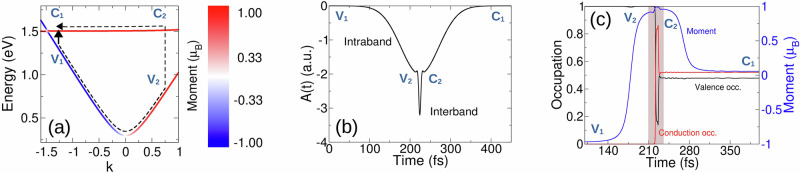
A model band structure exhibiting spin hybridization and the intraflip mechanism. **a** Two possible light induced excitations within a spin hybridized electronic structure are indicated: a direct optical excitation from valence to conduction, *V*_1_ → *C*_1_, and a second intraflip excitation indicated by the dashed lines *V*_1_ → *V*_2_ → *C*_2_ → *C*_1_. The former is forbidden as *V*_1_ and *C*_1_ have opposite spin. The intraflip excitation, however, involves first an intra-band evolution from spin down to spin up (*V*_1_ → *V*_2_) followed by an allowed spin up to spin up transition (*V*_2_ → *C*_2_). After intra-band evolution *C*_2_ → *C*_1_ this results in a spin flip excitation *V*_1_ → *C*_1_, mediated by intra-band evolution of crystal momentum. **b** The vector potential of the model pulse that generates this excitation pathway. **c** The change in occupation and moment during this intraflip process. Between *V*_1_ and *V*_2_ there is no change in occupation numbers—the valence band occupation is 1 and the conduction 0—while the spin moment rotates from down to up. This is followed by excitation to the conduction band, indicated by the shaded area, in which the conduction occupation rises to  ~ 0.5. Finally, in the second half cycle of the Gaussian pulse the moment reduces as the valence band state rotates back from up to down (along *V*_2_ → *V*_1_) while the conduction band moment remains up (*C*_2_ → *C*_1_). The overall effect is therefore a moment increase.

To demonstrate further the basis of the intraflip mechanism we now consider dynamical evolution within a minimal two band model consisting of a single (low energy) band in which spin up evolves to spin down, and a higher energy band that is pure spin up. This represents the simplest possible model realization of the spin hybridized valence sector and pure spin conduction sector of CrI_3_; the corresponding model band structure is shown in Fig. 6(a). We take the Fermi level to be at 1.4 eV, and thus *V*_1,2_ are valence states while *C*_1,2_ are conduction band states.

A direct optical excitation from valence to conduction, *V*_1_ → *C*_1_ as indicated by the full arrow, is forbidden as the states *V*_1_ and *C*_1_ are spin down and spin up respectively. An intraflip excitation pathway, indicated by the dashed line *V*_1_ → *V*_2_ → *C*_2_ → *C*_1_, is generated by the pulse shown in Fig. 6b. This consists of two Gaussian envelopes, the first of which, labeled “intraband", drives an evolution of momentum from *V*_1_ → *V*_2_ and back without generating inter-band transitions while the second, labeled “interband", generates inter-band excitation from *V*_2_ to *C*_2_. During the initial intra-band excitation valence and conduction occupation are fixed at 1 and 0 respectively, while the spin moment evolves from down to up, panel (c). At exactly half-cycle, the shaded region, excitation from valence to conduction occurs, allowed as both states are now spin up, with the conduction and valence occupancy then both, after excitation,  ~ 0.5. Finally, the second half-cycle returns the crystal momentum to its initial value, with the overall result a spin flip excitation *V*_1_ → *C*_1_.

The essence of the mechanism is thus in this way distilled into two processes of intra-band and inter-band evolution driven by the laser pulse. The intra-band momentum shifts imparted by the former allow a non-direct excitation, by-passing the restriction on excitation between orthogonal spin channels that holds for *δ***k** = **0** excitations. One should note that the intra-band excitation, by itself, has no overall effect on the system: an electric field applied to a full band does not, without electron-hole excitation, generate net change in the system. However the combination of strong intra-band motion with electron-hole excitation results, as described above, in dramatic new excitation pathways. The requirement of large (comparable to the Brillouin zone) intra-band evolution mandates a large vector potential amplitude, thus restricting this mechanism to the ultrafast regime for which such large amplitude pulses remain below the material damage threshold.

Having established the robustness of our result for CrI_3_ across distinct methodologies, we now consider the quite different material of CrSBr. Two simple rules of band design can be formulated for the intraflip mechanism of moment increase to *maximally* hold: (i) a spin hybridized valence band – necessary to induce the intra-band spin rotation – and (ii) spin split and spin pure conduction bands allowing the light pulse to be tuned to allow only spin up or spin down transitions. Evidently, this mechanism will also hold—albeit weakened—in the case of spin hybridization of the conduction bands, provided they exhibit sufficient imbalance of spin character with respect to the valence sector, such that net spin flips from valence to conduction will occur upon excitation.

**Fig. 7:**
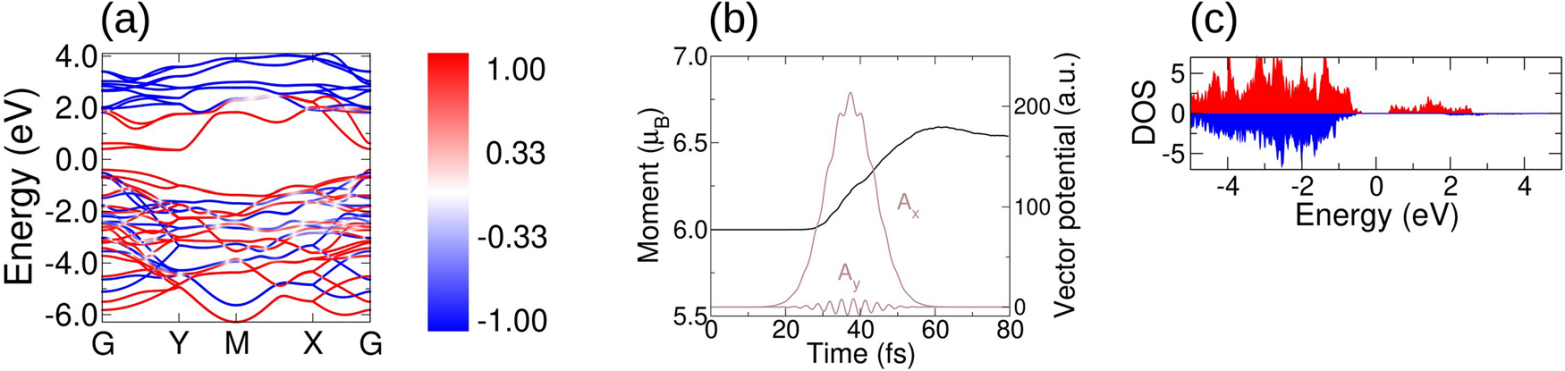
Ultrafast light-induced moment increase in CrBrS. **a** The band structure of CrSBr calculated in the Wannierized tight-binding method with band color indicating the magnitude of the *z*-component of spin (the *x* and *y* components are zero). **b** Action of a light pulse (vector potential shown in gray, peak electric field 3.7 V/nm and fluence 9.6 mJ/cm^2^) generates a moment increase similar to that seen in CrI_3_, with in (**c**) presented the TD-DOS after excitation by light (red and blue correspond to up and down spin channels respectively).

CrSBr meets both requirements (i) and (ii) above, as may be seen from the band structure presented in Fig. 7a. The conduction band splitting between the spin channels is, however, significantly reduced with the conduction majority and minority spin channels now overlapping. For this material we therefore employ a dual frequency (double pumped) pulse with a large amplitude linearly polarized THz component (duration 15.3 fs), designed to induce large intra-band evolution of momentum, and a circularly polarized infrared component (frequency 1.3 eV, duration again 15.3 fs) that induces inter-band transitions, pulse vector potential shown in gray in panel (b). Such “hencomb" pulse designs^42^ have proven to have utility in diverse contexts of light-matter control, including valley polarization of gapless graphene^43^, and generating pure spin and valley currents^44,45^. This pulse, as can be seen, generates a moment increase of 0.5*μ*_*B*_, falling to 0.4*μ*_*B*_ post pulse, also panel (b), with the transient density of states after the pulse shown in panel (c). This increase signals again the dominance of intraflip in the early time spin dynamics when the pulse energy is tuned from a valence spin hybridized band to a spin pure majority conduction band, highlighting the generality of the intraflip mechanism of light induced moment increase.

## Discussion

The fact that light couples directly to the momentum of the electron, but not to its spin, naturally invites the picture of a separation of time scales between these processes. Spin preserving charge excitation occurs effectively instantly with a strong laser pulse, in this picture, with spin-orbit induced spin flips occurring after some time delay. Our work presents a strikingly different picture: spin-orbit coupling generates a ground state spin texture in which spin direction is dependent on crystal momentum, implying that direct light pulse coupling to momentum is also a direct light pulse coupling to spin direction.

This forms the basis of an intraflip in which intra-band momentum evolution and inter-band transition combine to excite a valence band state $| {{{\bf{k}}}}\uparrow v\rangle$ to a conduction band state $| {{{\bf{k}}}}\downarrow c\rangle$. This process, as it is driven directly by the light pulse, occurs on a time scale dictated only by the pulse duration, and so can be activated on time scales from sub-femtosecond to hundreds of femtoseconds. Employing tight-binding and state-of-the-art first principles calculations of spin dynamics we have demonstrated massive light induced moment enhancement in the two dimensional magnets CrI_3_ (2*μ*_*B*_) and CrSBr (0.5*μ*_*B*_). The electronic structure required for the effect to occur is, however, general: a spin hybridized valence band and a spin pure up conduction band, implying that this effect will be found in other two- and three-dimensional semi-conducting/insulating magnetic materials. NiFe_2_O_4_ (nickel ferrite), possessing spin pure majority conduction bands with the valence sector exhibiting overlapping majority/minority bands represents an example of the latter. While attosecond pulses have theoretically uncovered a moment increase of  < 0.1*μ*_*B*_^46^ and light induced magnetic order supports a moment increase of  < 0.2*μ*_*B*_^47^, the lightwave control, magnitude, and robustness, of the magnetic enhancement found here is unprecedented. Our work thus opens up new avenues of control over magnetism, in which light pulses may effectuate full control of the magnetic moment, on time scales from a single to many optical cycles.

## Methods

### Tight-binding scheme

The general two-center tight-binding Hamiltonian is given by

1 $${H}_{0}={\sum }_{ij}{t}_{ij}{c}_{j}^{{{\dagger}} }{c}_{i}$$

where *t*_*ij*_ is the hopping amplitude between sites *i* and *j* (we suppress all other atomic indices). The *t*_*ij*_ are obtained via Wannierization using the Quantum Espresso package in conjunction with the Wannier90 software suite, both for CrI_3_ and CrSBr.

#### Numerical parameters for CrI_3_

We use a 10 × 10 × 1 k-mesh, a vacuum of 20 Å, and the LDA+U scheme for exchange correlation; *U* = 4.08 eV and *J* = 0.0 eV are employed which we find reproduces band structures obtained in the literature for this material. The unit cell parameters are *a* = *b* = 7.00 Å and *c* = 21.40 Å. We Wannierize the *d* bands of Cr and the *p* bands of I, yielding a 56 band Hamiltonian.

#### Numerical parameters for CrSBr

We use a 15 × 15 × 1 k-mesh, a vacuum of 20 Å, and the LDA scheme for exchange correlation; we find reproduces band structures obtained in the literature for this material. The unit cell parameters are *a* = 3.54 Å and *b* = 4.75 Å, *c* = 16.42 Å. We Wannierize the *d* bands of Cr and the *p* bands of S and Br, resulting in 44 bands in the Wannierization.

#### Tight-binding dynamics

This time dependent system ket can be expanded in a basis of Wannier states at crystal momentum **k**(*t*)

2 $$| {\Psi }_{{{{\bf{q}}}}}(t)\rangle={\sum }_{n}{c}_{n{{{\bf{q}}}}}(t)| {\Phi }_{n{{{\bf{k}}}}(t)}\rangle$$

with **k**(*t*) = **q** − **A**(*t*)/*c* given by the Bloch acceleration theorem (with **q** the crystal momentum at *t* = 0).

Dynamical evolution is governed by the time-dependent Schrödinger equation

3 $$i{\partial }_{t}{c}_{{{{\bf{q}}}}}(t)=H({{{\bf{k}}}}(t)){c}_{{{{\bf{q}}}}}(t)$$

where **k**(*t*) is given by the Bloch acceleration theorem.

### Time dependent density functional theory

Real-time TD-DFT ^48,49^ rigorously maps the computationally intractable problem of interacting electrons to a Kohn-Sham system of non-interacting electrons in an effective potential.

Time dependent density functional theory (TDDFT) is an ab-initio method for solving the dynamics of many-electron systems via a computationally tractable non-interacting system, known as the Kohn-Sham (KS) system. The non-interacting time-dependent Kohn-Sham (TDKS) equation for periodic systems reads:

4 $$\begin{array}{r}i\frac{\partial {\phi }_{j{{{\bf{k}}}}}({{{\bf{r}}}},t)}{\partial t}=\left[\frac{1}{2}{\left(-i{{{\boldsymbol{\nabla }}}}+\frac{1}{c}{{{{\bf{A}}}}}_{{{{\rm{ext}}}}}(t)\right)}^{2}+{v}_{{{{\rm{S}}}}}({{{\bf{r}}}},t)+\frac{1}{2c}{{{\boldsymbol{\sigma }}}}\cdot {{{{\bf{B}}}}}_{{{{\rm{S}}}}}({{{\bf{r}}}},t)\right. \\ \left.+\frac{1}{4{c}^{2}}{{{\boldsymbol{\sigma }}}}\cdot \left({{{\boldsymbol{\nabla }}}}{v}_{{{{\rm{S}}}}}({{{\bf{r}}}},t)\times -i{{{\boldsymbol{\nabla }}}}\right)\right]{\phi }_{j{{{\bf{k}}}}}({{{\bf{r}}}},t)\end{array}$$

where *ϕ*_*j***k**_(**r**, *t*) are two-component Pauli spinor TDKS orbitals with quasi-momentum **k,** **A**_ext_(*t*) is the external laser field, written as a purely time-dependent vector potential, ***σ*** are the Pauli matrices, *v*_S_(**r**, *t*) = *v*_ext_(**r**) + *v*_H_(**r**, *t*) + *v*_XC_(**r**, *t*) is the KS effective scalar potential, and **B**_S_(**r**, *t*) = **B**_ext_(**r**, *t*) + **B**_XC_(**r**, *t*) is the KS effective magnetic field. The external scalar potential, *v*_ext_(**r**), includes the electron-nuclei interaction, while **B**_ext_(**r**, *t*) is a external magnetic field which interacts with the electronic spins via the Zeeman interaction. The Hartree potential, *v*_H_(**r**, *t*) is the classical electrostatic interaction. Finally we have the XC potentials, the scalar *v*_XC_(**r**, *t*), and the XC magnetic field, **B**_XC_(**r**, *t*), which require approximation. In this work we used the adiabatic local density approximation (LDA).

#### Computational parameters for the TD-DFT calculations

In our calculations of CrI_3_ we employ a 10 × 10 × 1 k-mesh, 75 empty states corresponding to a energy cutoff of 70 eV, and the adiabatic local density approximation (LDA) as our exchange correlation functional *v*_*xc*_ and the time step is 2.4 attoseconds. The electronic temperature is set to 300 K. The unit cell dimensions are *a* = *b* = 6.89 Å and *c* = 20.0 Å.

## Supplementary information

Supplementary Information

Transparent Peer Review file

## Data Availability

The data generated in this study has been deposited in the Zenode database under accession code 10.5281/zenodo.19690545.

## Code availability

The Elk code is available at https://elk.sourceforge.io/. The tight-binding code at https://sourceforge.net/p/shallcross-big-pig-tb.
