# Supplementary material for: Giant magnetic moment increase by ultrafast laser light
Source: Nat Commun. 2026 Jun 3;17:5069. doi: 10.1038/s41467-026-73780-z (PMC13247070; doi:10.1038/s41467-026-73780-z)
Supplement: Supplementary file 1 — Supplementary Information [file 41467_2026_73780_MOESM1_ESM.pdf]

Giant magnetic moment increase by ultrafast laser light: Supplemental information

Sangeeta Sharma[1,2], Deepika Gill[1], Jyoti Krishna[1], Eddie Harris-Lee[3] John Kay Dewhurst[3], and Sam Shallcross[*,1]

[1]*Max-Born-Institut für Nichtlineare Optik und Kurzzeitspektroskopie, Max-Born-Strasse 2A, 12489 Berlin, Germany*

[2]*Institute for theoretical solid-state physics and Halle-Berlin-Regensburg Cluster of Excellence CCE, Freie Universität Berlin, Arnimallee 14, 14195 Berlin, Germany*

[3]*Max-Planck-Institut fur Mikrostrukturphysik Weinberg 2, D-06120 Halle, Germany*

1 One dimensional model of moment increase and decrease

We consider a four band one dimensional model Hamiltonian consisting of two strongly spin hybridized bands and two nearly spin pure conduction bands, shown in Fig. 1. Here the spin moment of each eigenstate is shown by the colour as indicated. The following Hamiltonian generates this band structure:

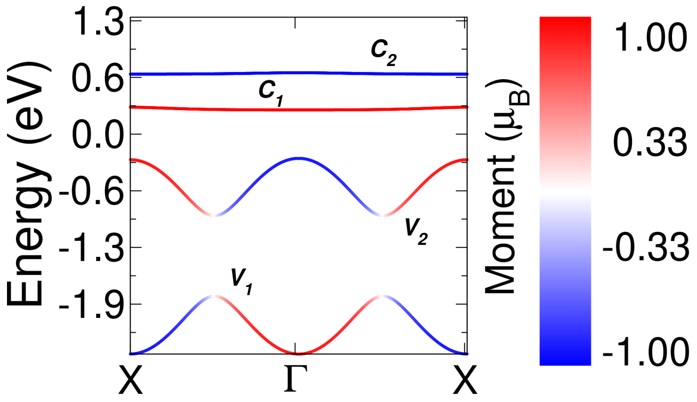

Supplementary Figure 1: *Model one dimensional band structure employed to illustrate the "intraflip" mechanism.* The four band model consists of two strolabelngly spin hybridized valence bands, labelled $v_{1,2}$, and two nearly pure spin conduction bands, $c_{1,2}$, with the Fermi level set to zero.

$$H = \begin{pmatrix} -\alpha_1(\alpha_2 + \cos(ak)) & \beta & \gamma & 0 \\ \beta & \alpha_1(\alpha_2 + \cos(ak)) & 0 & \gamma \\ \gamma & 0 & \alpha_3 + \alpha_5 \cos(ak) & 0 \\ 0 & \gamma & 0 & \alpha_4 + \alpha_5 \cos(ak) \end{pmatrix} \quad (1)$$

Here the order of the columns are v_1 (spin down valence), v_2 (spin up valence), c_1 (spin up conduction), and c_2 (spin down conduction); the diagonal elements therefore represent the energies of these bands, and the off diagonal elements their coupling with β controlling the valence band spin hybridization, and γ the coupling of valence to conduction. Parameter values that create the band structure shown in Fig. 1 can be found in Table 1.

This model captures the two key features of CrI_3 studied in the main manuscript: (i) strongly

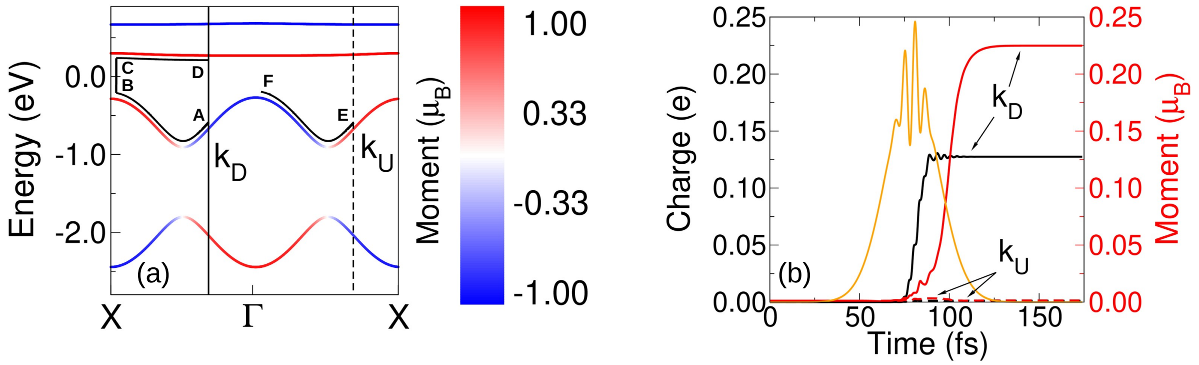

Supplementary Figure 2: *Intraflip mechanism of moment control.* (a) One dimensional model band structure designed to capture the key features of the CrI_3 electronic structure: spin hybridized valence bands and nearly spin pure and spin split conduction bands (the Fermi level is set to zero). (b) The charge and moment dynamics at two representative crystal momenta, denoted k_D and k_U in panel (a), with the pulse vector potential driving the dynamics indicated by the orange broken line. The dramatically different spin dynamics of these two momenta – excitation and significant increase in moment at k_D and almost no change at k_U – can be understood as the result of the two very distinct dynamical trajectories, indicated by ABCD and EF in panel (a). In the former case intra-band evolution of momenta rotates an initial spin down to spin up at the zone boundary (path AB), allowing optical excitation to the pure up conduction band, with the second pulse half cycle the returning this state to the initial momenta without further rotation, (path CD). The net result is a direct optical excitation A to D that has flipped the spin – an "intraflip transition". At k_U, by contrast, the intra-band evolution of momenta is accompanied by spin rotation from up to down (path EF), precluding direction excitation to the up conduction band, and resulting thus in almost no change from the ground state.

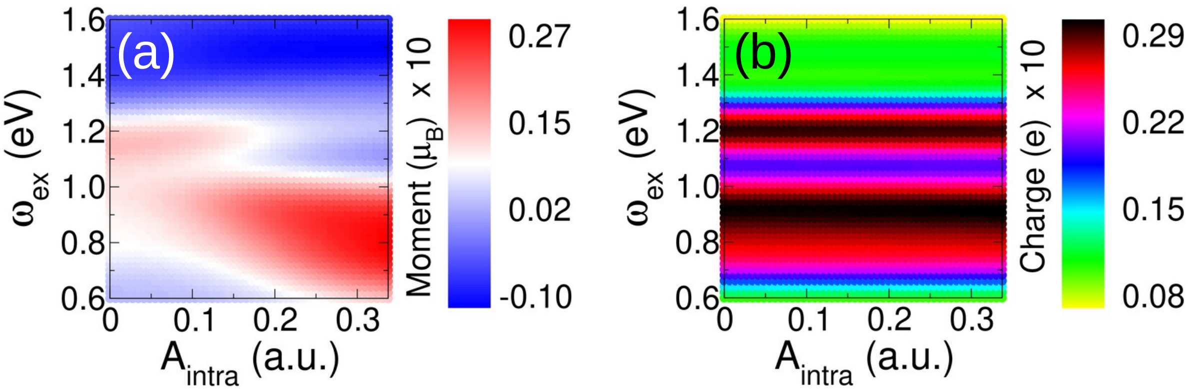

Supplementary Figure 3: *Role of pulse components in the intraflip mechanism.* (a) The change in the z-component of the spin moment upon light pulse excitation of the band structure shown in Fig. 1. The excitation laser pulse consists of a component whose frequency ω_{ex} is tuned through the conduction band, and a second sub-gap component of that induces only intraband evolution of momentum with pulse amplitude A_{intra}. The change in moment, plotted as a function of A_{intra} and ω_{ex} reveals (i) significant strengthening of the moment increase as A_{intra} is increased and (ii) a moment change that evolves from moment increase when ω_{ex} is in resonance with the spin up conduction band to moment decrease when ω_{ex} is in resonance with the spin down conduction band. In dramatic contrast to this spin physics in which both ω_{ex} and A_{intra} determine the moment change, the excited charge, panel (b), depends only on the excitation pulse component frequency, ω_{ex}.

spin hybridized valence bands and (ii) strongly spin split conduction bands that are pure up and pure down. We now consider two dynamical pathways within this band structure, at two representative crystal momenta of the excitation labelled k_D and k_U, Fig. 2(a). These pathways can be effectuated by a laser pulse consisting of a linearly polarized component resonant with the spin up conduction band augmented by a linearly polarized sub-gap component, vector potential shown in panel (b), a so-called "hencomb" pulse.

Parameter	Purpose	Value
α_1	Valence band width	1.00 eV
α_2	Valence band separation	0.00 eV
α_3	C_1 conduction band centre	1.60 eV
α_4	C_2 conduction band centre	2.00 eV
α_5	Conduction band width	0.00 eV
γ	Valence-conduction coupling	0.15 eV
β	Coupling of valence bands	0.45 eV

Supplementary Table 1: The parameters of the model Hamiltonian, Eq. 1, employed to demonstrate the intraflip concept. $\alpha_{1,4}$ determine band positions and widths, with β and γ the strength of, respectively, the coupling of the spin up and spin down valence bands v_1 and v_2, and the coupling of these valence bands to the conduction band system.

We first consider the crystal momenta k_D, focusing on the relevant valence band closest to the Fermi energy. The dynamical trajectory, i.e. the pathway of the light induced excitation in momentum-energy space, is schematically indicated by ABCD in Fig. 2(a). The leading edge of the pulse – dominated by the sub-gap component – generates intra-band evolution of momenta towards the Brillouin zone boundary (path segment AB) in turn evolving the spin from an initial down state at k_D to a spin up state at the zone boundary. The second pulse component, with energy tuned to the band gap, then generates inter-band excitation into the spin up conduction band, an allowed transition as spin is conserved. As this band manifold is pure up, the second half cycle of intra-band motion (path segment CD) does not further change the spin orientation. The net

result is thus an optical transition from valence to conduction at k_D that has flipped the spin. The dynamics of the excited charge (full black line) and m_z moment change (full red line) for this crystal momenta, panel (b), thus reveal an increase in both these quantities.

To bring out fully the role of the sub-gap and gap tuned components of the pulse employed in the manuscript to excite an increase in moment in the model, we present in Fig. 3(a,b) the change in magnetization and excited charge as a function of (i) the sub-gap component amplitude A_{intra} and (ii) the frequency of the gap tuned component ω_{ex}. The crucial role of the former in generating spin flips that allow moment increase can be seen by sending this component to zero: the moment enhancement reduces by a factor of $\sim$5. The excited charge, by contrast, remains almost independent of this pulse parameter.

2 Dependence of moment increase on pulse parameters

Here we consider the role of the amplitudes of the two pulse components of the “hencomb” pulse employed in the manuscript to CrSBr; the amplitude of the circularly polarized component A_{circ} and the moment of the linearly polarized component A_{intra}. We will apply this pulse waveform both to CrI_3 and CrSBr. A special case of this pulse waveform is $A_{intra} = 0$ in which the hencomb pulse reduces to a circularly polarized pulse. Employing the tight-binding method in Fig. 4 and 5 we present the moment as a function of A_{circ} for a series of values of A_{intra}, for CrI_3 and CrSBr respectively.

In Fig. 4 it can be seen that as the amplitude of A_{circ} increases the difference in the increase in moment excitation for different values of A_{intra} decreases: the circularly polarized pulse is “taking over” the work of the sub-gap linearly polarized component at large amplitude, generating itself sufficient intraband evolution of crystal momentum to drive the intarflip process. As can be seen, at the high amplitude end of A_{circ} an increase of more than 1 μ_B can be achieved by circularly polarized light acting alone.

A similar effect can be seen in CrSBr, in which a low A_{circ} the impact of the linearly polarized pulse is dramatic, but this reduces on increase of A_{circ}. However, driving the amplitude of the circularly polarized pulse to the high fluence limit results in excitation of charge to both conduction up and conduction down, with eventually the latter resulting in a decrease in moment from the ground state value of 6 μ_B.

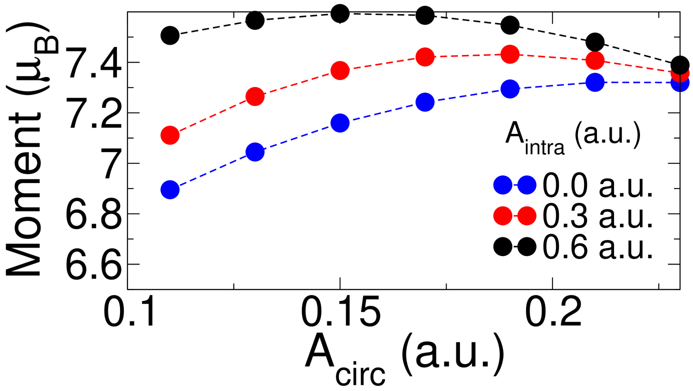

Supplementary Figure 4: *The laser excited ultrafast increase in moment of* CrI_3 *presented as a function of pulse component amplitudes; the ground state moment is 6.06*μ_B. We consider the laser pulse presented in Fig. 1(a) of the manuscript, consisting of a circularly polarized component tuned to be in resonance with the valence to spin up conduction band of CrI_3 of amplitude A_{circ}, and a linearly polarized sub-gap component of amplitude A_{intra}, generates a substantial increase of moment over that of the ground state. Increasing A_{circ} enhances the moment increase up to some critical value, after which non-linear processes generate charge excitation to both up and down conduction bands the light induced moment increase weakens. This is shown both for a pure circular pulse, $A_{intra} = 0$, as well as for two "hencomb" pulses of the type employed in the manuscript.

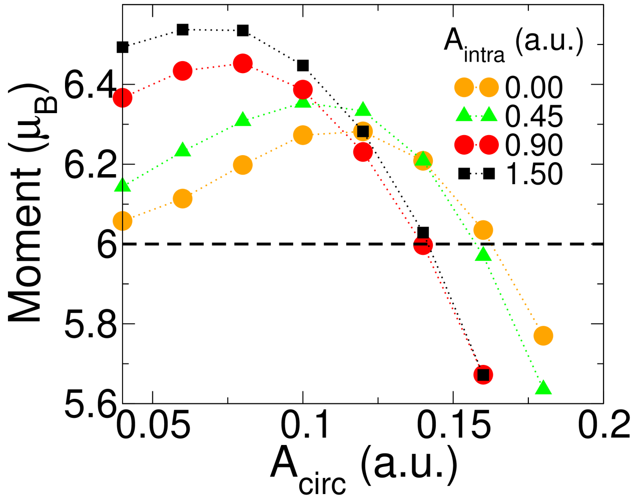

Supplementary Figure 5: *The laser excited ultrafast increase in moment of CrSBr presented as a function of pulse component amplitudes; the ground state moment is 6.0*μ_B*, indicated by the broken line.* As for CrI_3, Fig. 4, we present the moment variation with the amplitude of the spin up conduction band tuned circularly polarized pulse A_{circ}, for a series of values of the amplitude of the sub-gap linearly polarized component, A_{intra}.

3 Comparison of TD-DFT and tight-binding for linearly polarized pulses

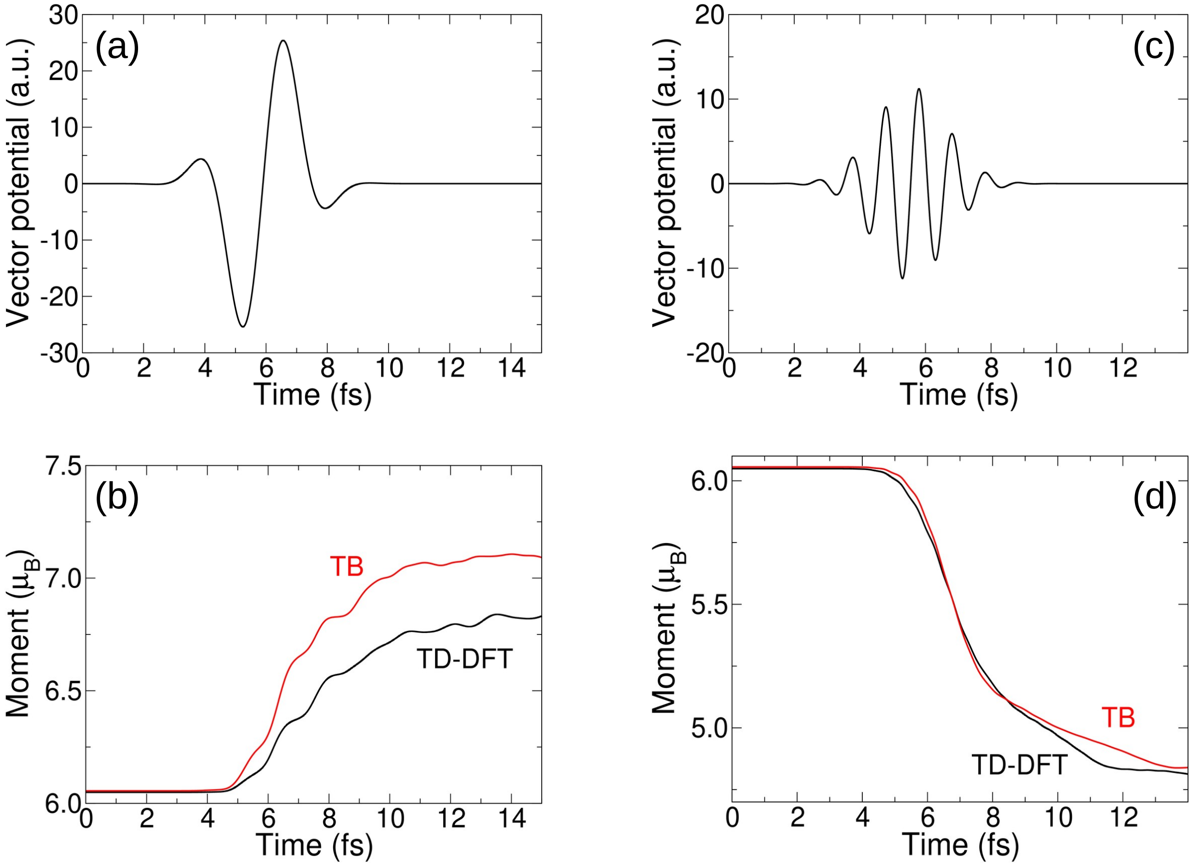

Supplementary Figure 6: *Comparison of time dependent density functional theory (TD-DFT) and tight-binding (TB) methods for calculating the transient moment.* (a) The vector potential of a linearly polarized pulse generating the moment *increase* shown in panel (b), and a higher frequency pulse, panel (c), that generates the moment *decrease* shown in panel (d). The transient moment is seen to be similar qualitatively similar behaviour when calculated by the TB and TD-DFT methods.

4 3-band model

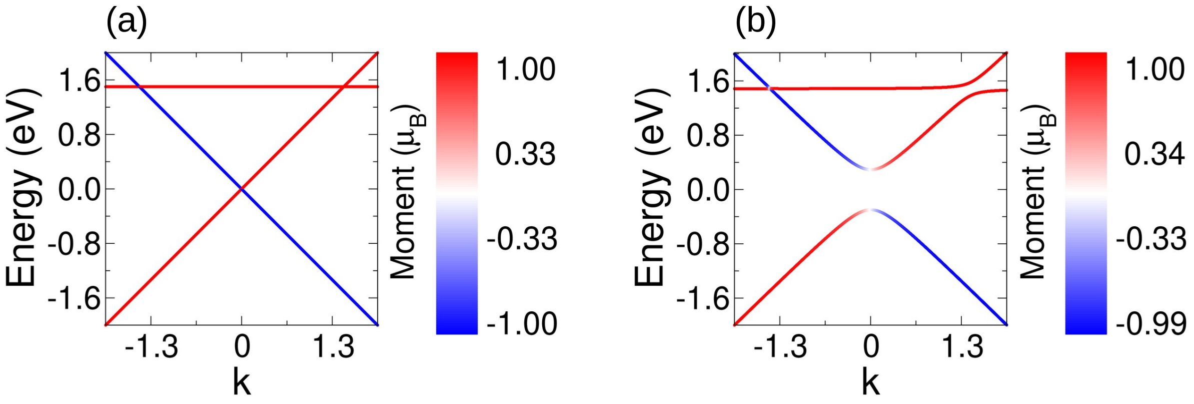

Supplementary Figure 7: *Model band structure*. (a) Band structure with $\Delta = \gamma = 0$ (b) Band structure for finite Δ and γ.

Intersecting spin up and down bands that hybridize via spin orbit-coupling generate bands that exhibit a continuous evolution between spin up and spin down states. This is illustrated in Fig. 7 via the 3-band Hamiltonian

$$H = \begin{pmatrix} -\alpha k & \Delta & 0 \\ \Delta & \alpha k & \gamma \\ 0 & \gamma & \epsilon \end{pmatrix} \quad (2)$$

where $\pm\alpha k$ represent two intersecting bands of opposite spin, with Δ a coupling between them. The high energy band at ϵ couples to these via the parameter γ. In panel (a) is shown the case of $\Delta = \gamma = 0$, i.e decoupled bands, and panel (b) the case in which these parameters are finite and both spin hybridization and coupling of these hybridized bands to the ϵ band can be seen.

This texture, as described in the main text, supports a three stage pathway that excites from a valence spin down state to conduction spin up state. This consists of: (i) a light driven intra-band evolution of momentum that rotates an initial spin down state to up, followed by (ii) inter-band excitation to the spin up conduction band, with (iii) subsequent intra-band evolution returning the state to its initial momentum while preserving the spin up state.

5 Attosecond moment enhancement

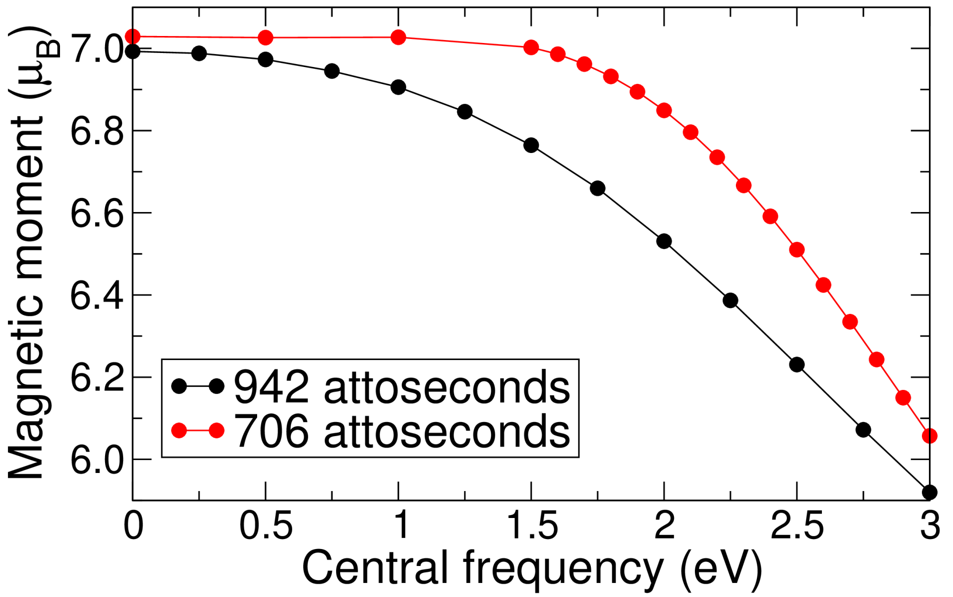

Supplementary Figure 8: *Light induced moment enhancement in CrI_3 in the attosecond regime*. By reducing the pulse central frequency the moment enhancement is continuously increased as intraflip excitations to the *minority* Cr conduction band are suppressed. Note shown here is the post-pulse moment; the ground state moment is 6.04 μ_B. As may be seen, for both a 942 and 706 attosecond pulse duration a substantial moment enhancement of 1 μ_B is obtained.

6 Comparison of band structures obtained via Elk and Quantum Espresso

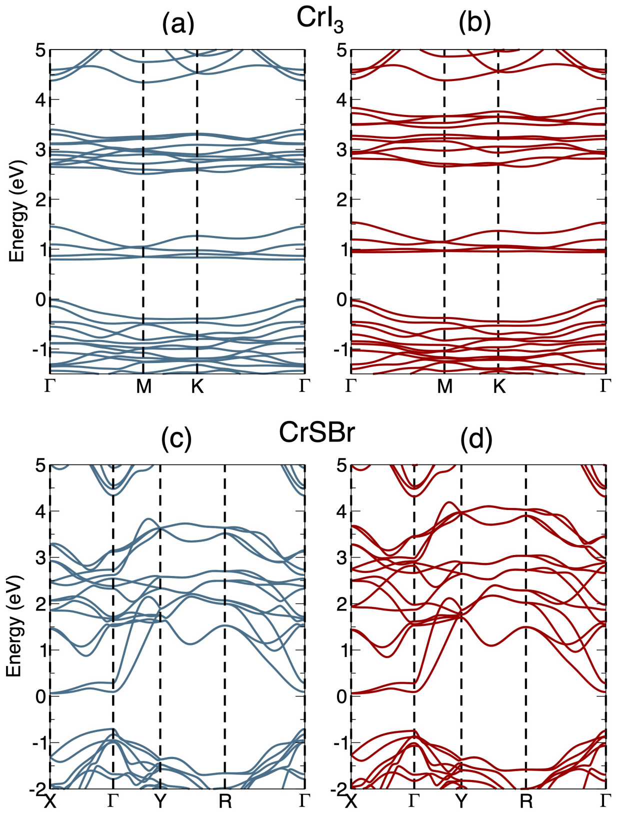

Supplementary Figure 9: *Band structures of CrI_3 and CrSBr.* Comparison of band structures obtained in the Elk and Quantum Espresso codes for CrI_3 (a,b) and CrSBr (c,d). The left hand panel is the band structure obtained via the Elk code, with the right hand panel that obtained via the Quantum Espresso code.

7 Origin of post-pulse oscillations in the spin moment

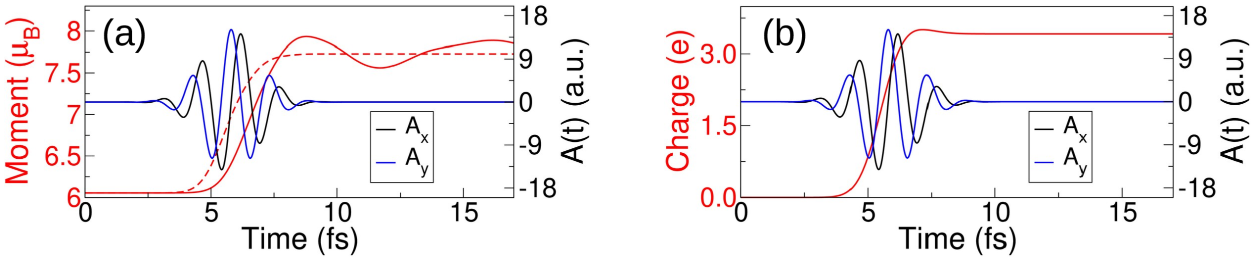

Supplementary Figure 10: (a) Intraband component of the magnetic moment, dashed line, along with the full magnetic moment (i.e. both intra- and inter-band components), full line. For temporal comparison the laser pulse that acts on the CrI_3 is shown also, referred to the right hand side axis. As can be seen the intra-band component, that depends on the occupancy of the band structure, remains unchanged after the pulse, with the full moment revealing oscillations about this value. These arise form interference between dynamical phases in the inter-band component of the moment, and do not reflect an excitation process. (b) The corresponding charge excitation which, just as for the intra-band component of the spin moment, is unchanged after the laser pulse.

Post laser-pulse the total spin moment is seen to vary; here we describe the origin of this post-pulse variation. To explore this we first note that the expectation value of the magnetic moment is

$$\mathbf{m}(t) = \sum_q \langle \Psi_{\mathbf{q}}(t) | \boldsymbol{\sigma} | \Psi_{\mathbf{q}}(t) \rangle \tag{3}$$

where $\Psi_{\mathbf{q}}$ is the time dependent wavefunction for a particular initial crystal momentum $\mathbf{q}$, and $\boldsymbol{\sigma}$ the vector of Pauli matrices $(\sigma_x, \sigma_y, \sigma_z)$. This time dependent wavefunction can be expressed in a

Houston basis as

$$|\Psi_{\mathbf{q}}(t)\rangle = \sum_n c_{n\mathbf{q}}(t) \left|\phi_{n\mathbf{k}(t)}\right\rangle \tag{4}$$

where $\left|\phi_{n\mathbf{k}(t)}\right\rangle$ are the local eigenstates at the time evolving k-vector $\mathbf{k}(t) = \mathbf{q} - \mathbf{A}/c$ that satisfy the ground state Hamiltonian $H_0 |\phi_{n\mathbf{k}}\rangle = \epsilon_{n\mathbf{k}}\phi_{n\mathbf{k}}$. Inserting Eq. 4 into Eq. 3 yields

$$\begin{aligned}
\mathbf{m}(t) &= \sum_{\mathbf{q}}\sum_n |c_{n\mathbf{q}}(t)|^2 \mathbf{m}_{n\mathbf{k}(t)} + \sum_{\mathbf{q}}\sum_{nn'} c^*_{n\mathbf{q}}(t) c_{n'\mathbf{q}}(t) \mathbf{m}_{nn'\mathbf{k}(t)} && (5)\\
&= \mathbf{m}_{intra} + \mathbf{m}_{inter} && (6)
\end{aligned}$$

where $\mathbf{m}_{n\mathbf{k}(t)} = \left\langle \phi_{n\mathbf{k}(t)} \middle| \boldsymbol{\sigma} \middle| \phi_{n\mathbf{k}(t)} \right\rangle$ and $\mathbf{m}_{nn'\mathbf{k}(t)} = \left\langle \phi_{n\mathbf{k}(t)} \middle| \boldsymbol{\sigma} \middle| \phi_{n'\mathbf{k}(t)} \right\rangle$. The first term is an intra-band term, summing only over a single band index and depending only on the occupation of the eigenstates $|c_{n\mathbf{q}}(t)|^2$, while the second term sums over both band indices n and n' and involves a product of expansion coefficients $c^*_{n\mathbf{q}}(t)c_{n'\mathbf{q}}(t)$. After the pulse the occupation numbers no longer change, and therefore the first term, the intra-band contribution to spin moment, is constant. The expansion coefficients are, however, time dependent post-pulse due to the dynamical phase factor $e^{i\epsilon_n t/\hbar}$, and thus the interband contribution to the spin moment of an excited state will in general exhibit time dependence.

The interband term can be re-written with the time dependence explicitly shown as

$$\mathbf{m}_{inter} = \sum_{\mathbf{q}} \sum_{nn'} e^{i(\eta_{n'\mathbf{q}} - \eta_{n\mathbf{q}})} e^{(\epsilon_{n\mathbf{q}} - \epsilon_{n\mathbf{q}})t/\hbar} \mathbf{m}_{nn'\mathbf{k}(t)} \tag{7}$$

where $\eta_{n\mathbf{q}}$ represent phases accumulated during the excitation process. Evidently this term retains a time dependence even after the pulse, driven by interference between the dynamical phases.

To demonstrate this numerically we calculate the intra- and inter-band contributions separately. To this end we consider the dynamics presented in Fig. 3 (g) of the manuscript, for which clear post-pulse variation of the total spin moment can be seen, and analyse the intra- and inter-band contributions to this. As can be see, Fig. 10(a), the intra-band spin moment (dashed line) is constant after the pulse.

The full moment, however, does change, and appears to oscillate about the intraband component of the magnetic moment, as one might expect from the structure of the intra- and inter-band terms in Eqs. 5-6 and 7. These post-pulse changes, therefore, occur solely due to quantum interference arising from the creation of superposition excited state by the laser pulse. They are thus driven by the inherent dynamical phases associated with the energies of the states contributing to this exited state, and do not reflect an excitation process. They are therefore a feature only of the very early few femtosecond regime in which such coherent states exist, and quantum decoherence processes will damp out these oscillations about the intraband component at longer times.

8 The “hencomb” pulse: example of altering the physics of excitation by intra-band transitions

The intra-flip mechanism described in the main paper represents a case in which the presence of intra-band evolution of momentum induced by the laser pulse dramatically alters the accompanying charge excitation. Here we describe a second example of this, in which the separation physics between intra- and inter-band transitions induced by the light pulse is particularly clear.

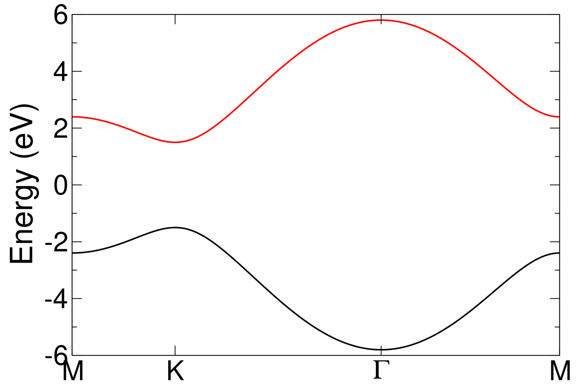

Supplementary Figure 11: *Band structure of gapped graphene with the K point gap set to 3 eV.*

Let us consider a minimal model of gapped graphene, a two band valley active system. The band structure of this system, with a gap of 3 eV, is shown in Fig. 11. Circularly polarized light excites charge at either the K or K* valleys depending on the helicity of the pulse, and this system thus represents the simplest model of the phenomena of light-valley coupling underpinning the field of valleytronics. Such light-wave excitations, however, cannot generate current: the excitation inherits the C_3 symmetry of the valley manifold and Bloch velocities cancel when integrated over momentum, leading to zero current after the pulse. This is shown in panels (a-c) of Fig. 12, where

in panel (a) we show the laser pulse vector potential, in panel (b) the induced current, which is zero after the pulse, and in panel (c) the momentum resolved charge excitation.

If we consider a linear THz pulse of large amplitude, vector potential shown in panel (d), then this cannot excite charge across that gap as the THz frequencies (i.e. meV energies) of the pulse fall far below the 3 eV energy difference of valence and conduction bands at the valley. The electric field of the pulse is, however, large due to the large vector potential amplitude, with a peak electric field of 2.5 MV/cm. Nevertheless, this pulse produces no physical effect: the current is zero after the pulse and no charge is excited, panels (e) and (f) respectively.

However, if we now at half cycle introduce the circularly polarized pulse described in panels (a-c), which yields an overall vector potential shown in panel (g), then the physics is dramatically different from either the circularly polarized pulse or the linear THz pulse: a large residual post-pulse current is now observed, panel (h), which occurs due to the shift off the valley centre of the charge excitation, panel (i). This post-pulse current arises as the cancellation of the Bloch velocities when integrated over the excited charge distribution can no longer occur due to the shift of the excitation off the valley centre. Thus by combining intraband motion (induced by the THz component) the the electron-hole excitation (induced by the circularly polarized component), the physical effect of the electron-hole excitation is dramatically altered.

Evidently, the application of a pulse can result in two processes: (i) an evolution of the crystal momentum and (ii) electron-hole excitation. What occurs in the excitation shown in Fig. 2 represents a combination of these two processes which can be described in three steps, as illustrated

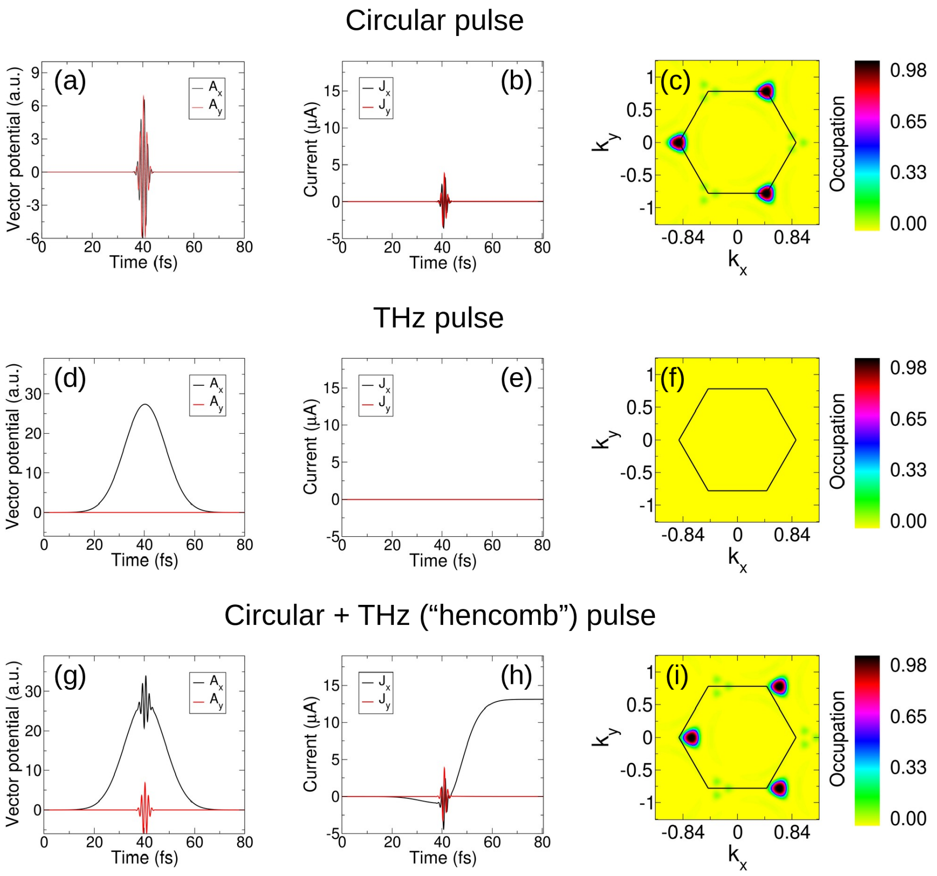

Supplementary Figure 12: *“Hencomb” excitation illustrating the role of a large electric field transient acting on a full band.* (a-c) A circularly polarized pulse applied to gapped graphene excites charge at one valley (in this case the K valley), but does not excite current as the C_3 symmetry of the charge excitation, inherited from the underlying valley manifold, results in complete cancellation of the microscopic current $\mathbf{j_k}$ when integrated over $\mathbf{k}$. (d-f) A Gaussian THz pulse can excite neither current or charge, as such a pulse acting on a full band generates no physical change. (g-i) The combination of these two pulses results in behaviour dramatically different from either acting alone with strong post-pulse current see, panel (i).

schematically Fig. 3 below. There are: (1) the THz pulse evolves a k-vector $\mathbf{k}_0$, distant from the valley centre K, to the valley centre at half cycle. (2) The circularly polarized pulse, tuned to the gap at K, drives charge across the gap, after which (3) the second half cycle of the THz pulse then evolves the crystal momentum back to the original momentum $\mathbf{k}_0$. The net result is then a charge excitation centered on $\mathbf{k}_0$, exactly as seen in panels (g-i) of Fig. 12.

This serves as an illustration of the fact that while a strong electric field transient generating intraband motion via the Bloch acceleration theorem acting alone cannot generate physical change, in combination electron-hole charge excitation it can result in dramatically different physics that the direct electron-hole charge excitation acting alone.
