# Supplementary material for: Giant magnetic moment increase by ultrafast laser light
Source: Nat Commun. 2026 Jun 3;17:5069. doi: 10.1038/s41467-026-73780-z (PMC13247070; doi:10.1038/s41467-026-73780-z)
Supplement: Supplementary file 2 — Transparent Peer Review file [file 41467_2026_73780_MOESM2_ESM.pdf]

nature portfolio

Peer Review File

Giant magnetic moment increase by ultrafast laser light

Corresponding Author: Dr Sam Shallcross

This file contains all reviewer reports in order by version, followed by all author rebuttals in order by version.

Version 0:

Reviewer comments:

Reviewer #1

(Remarks to the Author)
This is my second review while authors have changed the manuscript quite a lot and improved its quality. This work contains novelty about ultrafast increase of magnetic moment by femtosecond laser pulse. Authors performed time-dependent simulations using both tight-binding model and TD-DFT and indicates robust mechanism of ultrafast moment increase. Initially, I did not understand the intraband spin rotation, however in this revised manuscript, I am partially convinced by author's explanation. Many of explanations shown in main text are speculative, but for example in Fig. 9 of supplemental information authors provided partial evidence of this intraband spin flips. This should be included in the main text otherwise readers may feel that mechanism concluded in the main text is quite speculative. Is there any other way to observe this mechanism? Still, I do not fully understand this mechanism even author's plotted occupation and moment dynamics using two laser pulse [Fig. 9 of supplemental information]. It is also better to connect between simple theoretical model of the intraband spin flips and the calculation used in this study. Overall, I feel that this study presents novel light-induced increase of magnetic moment. So, this study should be published after addressing the points I mentioned above.

(Remarks on code availability)

Reviewer #2

(Remarks to the Author)
The authors report a significant and intriguing finding: an ultrafast enhancement of the magnetic moment induced by laser pulses in two representative two-dimensional magnetic van der Waals materials, CrI_3 and CrBrS. The phenomenon is analyzed using three complementary theoretical frameworks: (i) model-level calculations, (ii) Wannierized tight-binding models, and (iii) time-dependent density functional theory (TDDFT). The inclusion of both model-based and TDDFT approaches allows the authors to connect intuitive physical understanding with quantitative accuracy.

The proposed physical mechanism, a synergistic effect of optically driven transitions and intraband carrier motion, is both plausible and novel.

The robustness of the manuscript has improved thanks to the comments of previous reviewers and the appropriate revisions made by the authors.

The work is relevant to the communities working on ultrafast spin dynamics, light–matter interactions, and two-dimensional magnetic systems. Overall, the manuscript presents a solid and timely contribution with potentially broad impact. The authors' interpretation is convincing, and the results appear robust across the different theoretical treatments. The paper is generally well written and clearly structured.

As for the title, I recommend adding the word "magnetic" to better reflect the core focus of the study.

It would be valuable to include some discussion in the main text addressing the following points:

• Generality of the effect: Is this effect generic to all magnetic materials, or is it limited to 2D van der Waals magnets? The authors mention that it is generic; could they provide some examples of non-vdW 2D magnetic systems where similar phenomena could arise?

• Could this effect induce a magnetic moment in otherwise non-magnetic 2D vdW materials?
• How might this mechanism manifest in more complex 2D vdW magnets such as $Cr_2Ge_2Te_6$ (CGT) or Fe_3GeTe_2 (FGT)?
• The proposed excitation process involves sequential transitions between several electronic states. Can the authors comment on the factors determining the probability of this "intra(VB)–inter(VB–CB)–intra(CB)" absorption process? A clear definition of the model Hamiltonians describing the interaction between electrons (with their spin character) and photons would be beneficial to convey a more precise conceptual understanding of the light–matter interaction.
• The authors state that the same effect can be expected in CrSBr, despite finite spin hybridization in the conduction band. Could the statement in lines 42–44 be generalized to indicate that a finite increase of the magnetic moment can be expected when spin hybridization is stronger in the valence band than in the conduction band? It seems that an imbalance of spin mixing between valence and conduction bands could be a sufficient condition.
• Does the conduction band really need to be "spin-pure"? It seems that an imbalance between spin-up and spin-down transition probabilities may be sufficient for the mechanism to operate, provided that spin hybridization is stronger in the valence band than in the conduction band.
• How is the gained spin angular momentum compensated in this picture? It would be valuable to see some explicit discussion or numerical evidence addressing conservation of total angular momentum, i.e., whether an increase in spin angular momentum is accompanied by a corresponding decrease in orbital angular momentum. The current discussion, including exchanges with previous reviewers, does not fully resolve this point.
• While the reported results are compelling, the manuscript would benefit from a clearer identification of the precise physical mechanisms underlying the simulated phenomena. At present, the discussion feels somewhat descriptive. A deeper explanation of the physical principles governing the various scattering and absorption processes, and of why these processes are both allowed and not dominated by simple $\Delta k = 0$ direct interband transitions, would considerably strengthen the conceptual impact of the paper.
• Typographical corrections: Please correct typographical errors throughout the text. For example:"Indeed, several recent experimental works have demonstrated light-induced spin switching in CrI_3[27–29], showing that the material represents a practical experimental platform for investigating ultrafast spin dynamics."
• Line 73: "transitions from valence (v) to conduction (c)" — please use the same notation as in Fig. 2, namely valence band (VB) and conduction band (CB).
• Line 199: "light" is misspelled as lihgth.

(Remarks on code availability)

Reviewer #3

(Remarks to the Author)
Report

In the present theoretical work, the Authors showed that the absorption of ultrashort laser pulses in the ferromagnetic semiconductors CrI3 and CrSBr can lead to both an increase and a decrease in the magnetic moment, depending on the frequency of the laser. Special emphasis was placed on the increase in the moment, because this effect is as yet relatively unexplored. Two different approaches were chosen as methods: a tight-binding model and time dependent density functional theory, which both show similar dynamics of the magnetization and thus underpin the validity of the results. A central claim made by the authors is that they had identified a novel so-called "intraflip mechanism", which can explain the laser-induced enhancement and decrease of the magnetic moment. In fact, the question about the physical mechanism is of fundamental interest. Therefore, I would generally assess that the submitted work could represent a significant advance in its field of research and that it would also be of considerable interest to a broad spectrum of physicists working on ultrafast dynamics. However, even in the resubmitted paper, I still see some significant unclarities in the interpretation and discussion of the mechanism. Therefore, I cannot recommend publication in Nature Communications unless the Authors have clarified these ambiguities. Before explaining the problematic points in detail, let me comment on the Authors' responses to the Referees' criticisms.

The responses to the first Reviewer's criticisms appear convincing. The Authors now explicitly mention that their calculations lead to similar results with both linearly and circularly polarized light. Because this shows that a direct transfer of angular momentum from light to the spin system is not essential, the physical conclusions on the mechanism become more precise. The other concerns of the first Reviewer have also been taken into account. For instance, calculations of the XMCD response function and more details of the laser pulse as well as of the material properties are now included in the revised version.

Concerning the fundamental objection raised by the second Reviewer, namely that too few materials were considered in this theoretical work, I do not share his opinion. On the contrary one should be aware that the Authors' main goal is not to highlight material-specific differences but to investigate and to understand the fundamental physics behind the effect. For this purpose, considering only two examples and a simple model, as presented by the Authors, seems to me to be entirely sufficient. Further comments of the second Reviewer were also addressed convincingly by the Authors.

However, in my opinion, the responses to the third Reviewer's comments were not always convincing. The first point raised by this Referee was that an electrical field applied to the fully occupied valence band cannot cause any change to the system unless an electron has been excited to the conduction band. Unfortunately, the Authors' response that the effect of

Bloch acceleration on the electrons during the first half of the cycle and the effect during the second half of the cycle cancel each other out seems not applicable, as long as no electron-hole excitation has been generated, because there exists only a single many-electron state that describes the completely filled valence band (filled Fermi sea) which cannot change. In any case, I share the Reviewer's expectation that it is only through electron-hole excitation that a dynamic process is initiated, whereby only thereafter the hole in the valence band can change its wave vector and spin due to the Bloch acceleration effect. This physical picture is supported by Figure 2(b) of the Supplemental Materials, which shows that the excited charge in the conduction band increases faster than the total spin moment. Importantly, according to this physical picture, the sequence of microscopic processes is exactly the opposite of that claimed by the Authors (who proposed that first the electron in the filled valence band changes its spin and then, secondly, it is excited to the conduction band). Therefore, this point needs to be clarified. Either a truly convincing argument is required by the Authors or a reformulation of their interpretation.

The next issue raised by the third Reviewer concerns the question of which term in the Hamiltonian is responsible for the change in angular momentum during the enhancement of the total spin moment. Here, I agree with the Authors' answer that the spin angular momentum is transferred to orbital angular momentum (by means of spin-orbit coupling), which then in turn is transferred to the lattice. However, their remark in the manuscript "the present TD-DFT calculations we do not include the dynamics of the lattice degrees of freedom, and thus the flow of spin angular momentum, via orbital degrees of freedom, to the lattice is not included in our calculations" seems a little too brief to me. It is understandable that consideration of nuclear degrees of freedom would go beyond the scope of this work. However, it would certainly be helpful for the Reader if the Authors could discuss in more detail the different processes which might be relevant for that angular momentum flow from the spins to the lattice. The present explanation, that the changes in the spin moment take place basically during the Bloch acceleration of electrons, does not provide transparent insights into the involved microscopic processes.

In the following, I would like to make a few more comments in addition to the points already mentioned by the previous Reviewers.

i)
The intraflip mechanism has been presented by the Authors as if it were a novel mechanism. However, it is essentially based on a purely electronic Hamiltonian including the electronic bands and the spin-orbit interaction. These processes have not been considered for the first time but have already been used to explain conventional laser-induced demagnetization in metals. For this reason, I would consider it somewhat exaggerated to present the intraflip mechanism as a different mechanism. However, to my knowledge, its application to ferromagnetic semiconductors with their characteristic band structure is new and, therefore, worthy of publication.

ii)
In the discussion the Authors claimed that the light pulse directly couples to the spin direction through the light pulse coupling to momentum, and that the intraflip effect occurs on a time scale dictated only by the pulse duration. These statements seem somewhat misleading, as they would imply that spin flips occur exclusively during the pulse. However, this is not always strictly true. For example, Figures 3(c), 3(g), and 4(g) show that the magnetic moment continues to change even after the pulse has ended. Of course, the change in the moment is then only slight, but not vanishing. From a theoretical perspective, the interaction of the electrons with the laser (which is independent of spin) and the spin-orbit interaction are actually independent of each other. In the present work, systems with a very strong spin-orbit coupling were considered. See, for example, beta = 0.45 eV in Table 1 of the Suppl. Materials. Could this be the reason why the magnetic moment decreases during the same time scale as the laser pulse acts, and therefore the intraflip effect seems to be induced by a direct coupling between electrons and the pulse? It would be clarifying for the Reader if the Authors would formulate more precisely their statement. Moreover, could the Authors share their expectations regarding how the results would be for systems with weaker spin-orbit coupling?

iii)
A minor comment: The two panels (b) and (c) of Figure 1 contain a full and a dotted curve, where the meaning of the dotted curves remains unclear. For the panel (c) it is written that the dotted curve illustrates the intra-band contribution to the magnetic moment. What is this? Does this refer to the contribution from the valence band? The Authors should explain their numerical results and discuss the physical implications.

In conclusion, I recommend that the Authors clarify convincingly the above ambiguities. Without a corresponding revision, I cannot recommend publication in Nature Communications.

(Remarks on code availability)

Reviewer #4

(Remarks to the Author)
I co-reviewed this manuscript with one of the reviewers who provided the listed reports. This is part of the Nature Communications initiative to facilitate training in peer review and to provide appropriate recognition for Early Career Researchers who co-review manuscripts.

(Remarks on code availability)

Version 1:

Reviewer comments:

Reviewer #2

(Remarks to the Author)
The authors have significantly improved the quality of the manuscript by including a detailed description of the interactions involved in the proposed mechanism. The discussion of angular momentum conservation is convincing, the limitations of the model are clearly identified and do not significantly limit the aplicability. As stated before, we believe the article is novel and of high interest to the community of ultrafast magnetization dynamics. At this point, we can recommend the manuscript for publication.

(Remarks on code availability)

Reviewer #3

(Remarks to the Author)
The present work reports on laser-induced magnetic moment increase in ferromagnetic semiconductors. As part of this, the authors identify a novel spin-flip mechanism, the so-called intraflip mechanism. Therefore, the submitted work is of considerable interest to the research field of ultrafast spin dynamics. This is my second review of this paper, in which I now recommend that the submitted paper be published. The concerns expressed in the first report have been addressed, and as a result, the physical mechanism proposed by the Authors now appears clearer in the paper, from my point of view. In particular, I would like to mention that the insertion of the two new sections 4 (“Angular momentum conservation”) and 5 (“Model illustration of the intraflip mechanism”) has contributed to clarification. For these reasons, the resubmitted paper seems suitable for publication in Nature Communications.

(Remarks on code availability)

Reviewer #4

(Remarks to the Author)
I co-reviewed this manuscript with one of the reviewers who provided the listed reports. This is part of the Nature Communications initiative to facilitate training in peer review and to provide appropriate recognition for Early Career Researchers who co-review manuscripts.

(Remarks on code availability)

Open Access This Peer Review File is licensed under a Creative Commons Attribution 4.0 International License, which permits use, sharing, adaptation, distribution and reproduction in any medium or format, as long as you give appropriate credit to the original author(s) and the source, provide a link to the Creative Commons license, and indicate if changes were made.
In cases where reviewers are anonymous, credit should be given to 'Anonymous Referee' and the source.
The images or other third party material in this Peer Review File are included in the article's Creative Commons license, unless indicated otherwise in a credit line to the material. If material is not included in the article's Creative Commons license and your intended use is not permitted by statutory regulation or exceeds the permitted use, you will need to obtain permission directly from the copyright holder.
To view a copy of this license, visit https://creativecommons.org/licenses/by/4.0/

Reviewer #2 (Remarks to the Author):

This is my second review while authors have changed the manuscript quite a lot and improved its quality. This work contains novelty about ultrafast increase of magnetic moment by femtosecond laser pulse. Authors performed time-dependent simulations using both tight-binding model and TD-DFT and indicates robust mechanism of ultrafast moment increase. Initially, I did not understand the intraband spin rotation, however in this revised manuscript, I am partially convinced by author's explanation. Many of explanations shown in main text are speculative, but for example in Fig. 9 of supplemental information authors provided partial evidence of this intraband spin flips. This should be included in the main text otherwise readers may feel that mechanism concluded in the main text is quite speculative.

We have now included this as Fig. 5 of the manuscript with an associated new section "Model illustration of the intraflip mechanism". We agree with the Referee that this – while a model band structure – directly illustrates the "intraflip" mechanism and is thus valuable to include in the main manuscript. We thank the Referee for suggesting this.

Is there any other way to observe this mechanism? Still, I do not fully understand this mechanism even author's plotted occupation and moment dynamics using two laser pulse [Fig. 9 of supplemental information]. It is also better to connect between simple theoretical model of the intraband spin flips and the calculation used in this study.

We have made the connection; please see lines 187-215.

Overall, I feel that this study presents novel light-induced increase of magnetic moment. So, this study should be published after addressing the points I mentioned above.

We thank the Referee for careful reading of the paper and are pleased that the Referee agrees that the paper is now strengthened by the previous round of comments and is now suitable for publication.

Reviewer #4 (Remarks to the Author):

The authors report a significant and intriguing finding: an ultrafast enhancement of the magnetic moment induced by laser pulses in two representative two-dimensional magnetic van der Waals materials, CrI_3 and CrBrS. The phenomenon is analyzed using three complementary theoretical frameworks: (i) model-level calculations, (ii) Wannierized tight-binding models, and (iii) time-dependent density functional theory (TDDFT). The inclusion of both model-based and TDDFT approaches allows the authors to connect intuitive physical understanding with quantitative accuracy.

The proposed physical mechanism, a synergistic effect of optically driven transitions and intraband carrier motion, is both plausible and novel.

The robustness of the manuscript has improved thanks to the comments of previous reviewers and the appropriate revisions made by the authors.

The work is relevant to the communities working on ultrafast spin dynamics, light–matter interactions, and two-dimensional magnetic systems. Overall, the manuscript presents a solid and timely contribution with potentially broad impact. The authors' interpretation is convincing, and the results appear robust across the different theoretical treatments. The paper is generally well written and clearly structured.

We thank the Referee for the careful reading of the manuscript and the several suggestions for improvements of the presentation and orientation enumerated below, in particular the suggestion to more carefully analyze the question of angular momentum conservation, and the prompt to include a more detailed discussion of possible 3d and spin compensated materials in which this effect may be seen.

As for the title, I recommend adding the word "magnetic" to better reflect the core focus of the study.

We have changed the title replacing "moment" with "magnetic moment".

It would be valuable to include some discussion in the main text addressing the following points:

• Generality of the effect: Is this effect generic to all magnetic materials, or is it limited to 2D van der Waals magnets? The authors mention that it is generic; could they provide some examples of non-vdW 2D magnetic systems where similar phenomena could arise?

An example of non-vdW 2d magnetic systems would be 3d bulk insulators in which the exchange splitting and crystal field act to create a band structure in which the valance bands are spin hybridized, while the conduction band exhibit significantly less pronounced spin hybridization. A potential example of this is Yttrium iron garnet in which there are significant regions of spin pure conduction bands while the valence sector feature overlapping spin bands[1] (although in this case as the pure spin region is minority it would lead to ultrafast demagnetization). A second example would be $NiFe_2O_4$ ferrite which has a spin pure majority conduction bands with the valence sector again possessing overlapping majority/minority bands[2]; in this case the materials appears to be a perfect 3d insulator for the "intraflip" effect with the potential for an ultrafast increase in magnetic moment, however Ref. 2 appears to be (as is Ref. 1) a scalar relativistic calculation in which spin hybridization is not considered. We plan to investigate these two materials in subsequent work as potential 3d magnetic insulator cases of "intraflip" driven ultrafast change in moment, and thank the Referee for the suggestion to provide concrete examples of systems beyond vdW 2d magnetic

materials in which such physics may be seen. We have now included these examples in the discussion, see lines 253-255.

• Could this effect induce a magnetic moment in otherwise non-magnetic 2D vdW materials?

This is an intriguing possibility and we speculate it may be so in the case of 2d vdW Rashba systems, such as, for example, GeTe[3]. In this case, however, as both conduction and valence sectors have paired bands with approximately opposite moment split only be 10-50 meV Rashba splitting this would require careful tuning of the pulse parameters and longer duration pulses (such that their frequency components are dominated in a 10s of meV spectral range).

• How might this mechanism manifest in more complex 2D vdW magnets such as $Cr_2Ge_2Te_6$ (CGT) or Fe_3GeTe_2 (FGT)?

The key difference here is that these are metallic systems and so the light induced excitations to occur from all energies $E_1 < E_F$ and $E_2 > E_F$ for which $E_1 - E_2$ has a sufficient frequency component in the laser pulse. This complicates the pulse design but does not, in our view, exclude metallic systems such as CGT and FGT from consideration of this effect.

• The proposed excitation process involves sequential transitions between several electronic states. Can the authors comment on the factors determining the probability of this "intra(VB)–inter(VB–CB)–intra(CB)".

The inter-band transitions are dominated by the dipole matrix elements of the transition, which the intraband transitions are determined by the vector potential amplitude. It is this latter point that fixes the proposed mechanism to the ultrafast regime: only at femtosecond duration do large amplitude vector potential laser pulse possess a fluence below the damage threshold of a 2d (or 3d) material. We have now mentioned this, see lines 213-215.

• The authors state that the same effect can be expected in CrSBr, despite finite spin hybridization in the conduction band. Could the statement in lines 42–44 be generalized to indicate that a finite increase of the magnetic moment can be expected when spin hybridization is stronger in the valence band than in the conduction band? It seems that an imbalance of spin mixing between valence and conduction bands could be a sufficient condition.

Yes we agree with the Referee; we expand on this point in reply to the related query below.

• Does the conduction band really need to be "spin-pure"? It seems that an imbalance between spin-up and spin-down transition probabilities may be sufficient for the mechanism to operate, provided that spin hybridization is stronger in the valence band than in the conduction band.

Yes, this is correct and we have added a sentence to the paper to state this (see lines 222-224).

CrSBr is in fact exactly such a case: the conduction band does exhibit spin hybridization, but this is significantly less that in the valence band, allowing the effect to occur.

• How is the gained spin angular momentum compensated in this picture? It would be valuable to see some explicit discussion or numerical evidence addressing conservation of total angular momentum, i.e., whether an increase in spin angular momentum is accompanied by a corresponding decrease in orbital angular momentum. The current discussion, including exchanges with previous reviewers, does not fully resolve this point.

The total angular momentum i.e. nuclear angular momentum ($\hat{N}$), and the orbital ($\hat{L}$) and spin ($\hat{S}$) angular momentum of the electron system is, of course, a conserved quantity as the total momentum operator $\hat{N}+\hat{L}+\hat{S}$ commutes with our Hamiltonian. However, in the present work the nuclear degrees of freedom are clamped, and thus we cannot explore the full physics of angular momentum and its conservation. However, we can, as the Referee suggests, examine the dynamics of the spin and orbital angular momentum separately with a view to at least probing further into the physics underlying orbital angular momentum conservation.

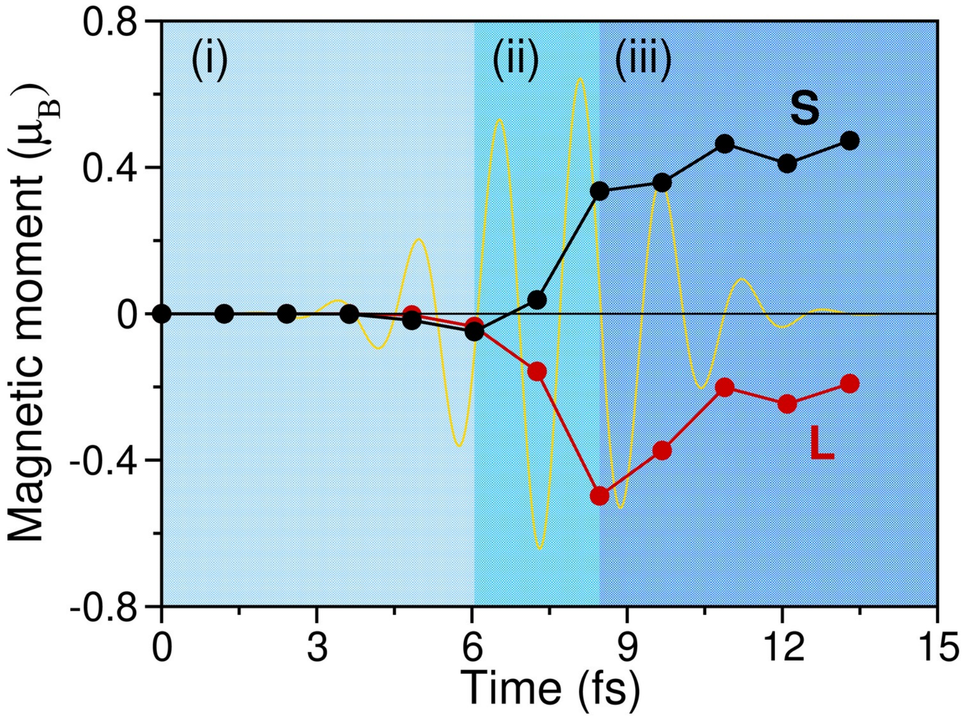

Fig. 1: *Early time spin and orbital angular momentum dynamics*. Shown are the temporal change in spin $\mathbf{S}$ and orbital angular momentum $\mathbf{L}=\mathbf{r}\times\mathbf{p}$, both evaluated within the Cr muffin-tin, along with the laser pulse shown in background (grey). Three regimes of spin and angular momentum dynamics can be discerned. (i) At early times both $\mathbf{L}$ and $\mathbf{S}$ exhibit a reduction, behaviour similar to that seen in demagnetization dynamics of elemental Ni and Co. (ii) This, however, is succeeded by a regime in which the spin angular momentum increases – generated by "intraflips" associated with the intraband evolution of momentum – while the orbital angular momentum decreases. The microscopic muffin-tin currents associated with this reduction in L would, via forces acting on the nuclei, transfer this reduction in angular momentum to the lattice, thus ensuring conservation of angular momentum.

We should stress at the outset that there are fundamental theoretical difficulties that remain outstanding unsolved challenges in the ultrafast condensed matter community with regard to the analysis of angular momentum, namely that as the position operator is undefined in a periodic solid (the operator $\mathbf{r}$ does not respect the crystal transition symmetry), then operator for orbital angular momentum, $\mathbf{L}=\mathbf{r}\times\mathbf{p}$ is also not well defined for a solid. This situation is resolved at *equilibrium* by the "modern theory of orbital magnetism" in which, either employing a Wannier basis or semi-classical approach, one has the expression

$$\mathbf{L}=\sum_n \langle\boldsymbol{\nabla}_{\mathbf{k}}u_{n\mathbf{k}}|(H_{\mathbf{k}}+E_{n\mathbf{k}})|\nabla_{\mathbf{k}}u_{n\mathbf{k}}\rangle \qquad \text{(Equation 1)}$$

where $|u_{n\mathbf{k}}\rangle$ is the periodic part of the Bloch function $|\psi_{n\mathbf{k}}\rangle=e^{i\mathbf{k}.\mathbf{r}}\,|u_{n\mathbf{k}}\rangle$ with $H_{\mathbf{k}}=e^{-i\mathbf{k}.\mathbf{r}}He^{i\mathbf{k}.\mathbf{r}}$ and $H_{\mathbf{k}}\,|u_{n\mathbf{k}}\rangle=E_{n\mathbf{k}}\,|u_{n\mathbf{k}}\rangle$.

However, there is no corresponding expression for the non-equilibrium situation, and thus $\mathbf{L}(t)$ cannot at present be calculated for a periodic solid. This remains a fundamental outstanding theoretical challenge, as yet unresolved.

To bypass this we can, however, calculate $\mathbf{L} = \mathbf{r} \times \mathbf{p}$ within the muffin-tin (i.e. a sphere placed around the atomic centre). We have previously employed this approach to study the angular momentum redistribution in the elemental magnets Ni and Co, and have utilized this here, calculating $\mathbf{L}(t)$ and $\mathbf{S}(t)$ within the Cr muffin-tin, shown here as Fig. 1 of this reply.

This reveals three distinct temporal domains, indicated by the background colour of the plot:

(i) At early times before the peak of the pulse spin and angular momentum both begin very slightly to decrease, behaviour that is reminiscent of the demagnetization dynamics of Ni and Co.

(ii) The "intraflip" mechanism then takes over, active at the peak of the pulse as this requires a large amplitude of the vector potential to drive intra-band evolution of crystal momentum, generating an increase in $\mathbf{S}(t)$. This increase in $\mathbf{S}(t)$ however is accompanied by a corresponding *reduction* in orbital angular momentum $\mathbf{L}(t)$ (note the plot in Fig. 1 shows the change in these quantities). The dynamical excitation thus has generated microscopic currents within the muffin-tin that to some extent compensate for the increase in $\mathbf{S}(t)$. These microscopic currents generate forces on the nuclei, which would then, if the nuclei were not clamped, induce nuclear dynamic transferring angular momentum from the $\mathbf{L}$ of the electron system to the lattice. Thus ultimately the increase in $\mathbf{S}$ would be compensated for by a decrease in the angular momentum of the nuclear system.

(iii) Finally we see that $\mathbf{L}$(t) begins to increase, as these forces have acted on the clamped nuclei, while the spin continues to increase somewhat.

This provides a sketch of the microscopic dynamical pathway by which the increase in spin angular momentum will ultimately be compensated by the lattice. This provides insight into the microscopic dynamical pathway by which the increase in spin angular momentum will ultimately be compensated by the lattice, and have therefore added Fig. 1 and this discussion as new section of the manuscript (Section 4, lines 162-186). We thank the Referee for encouraging us to provide a microscopic underpinning of the dynamics of $\mathbf{S}$, $\mathbf{L}$, and the lattice, and feel that by including this our paper may inspire further work on this outstanding problem.

• While the reported results are compelling, the manuscript would benefit from a clearer identification of the precise physical mechanisms underlying the simulated phenomena. At present, the discussion feels somewhat descriptive. A deeper explanation of the physical principles governing the various scattering and absorption processes, and of why these processes are both allowed and not dominated by simple Δk = 0 direct interband transitions, would considerably strengthen the conceptual impact of the paper.

Following also the suggestion of Referee #2 we have added a new section in which the the proposed mechanism is illustrated in a spin hybridized model band structure (see lines 187-215, in particular lines 206-125). The excitation is now driven by a pulse of two components, one generating intraband transitions and one interband transitions. In this way, the role of these two types of excitation can be transparently seen. We have in this section also described how the role of intraband transitions – i.e. for the physics not to be dominated by the $\Delta\mathbf{k} = \mathbf{0}$ transitions – requires large vector potential amplitudes characteristic of the ultrafast regime. In short, the mechanism requires the vector potential amplitude $\mathbf{A}_0/c$ to be comparable to the scale to the reciprocal lattice vectors for the effect to be significant, and for this to occur requires femtosecond scale pulses for the fluence to be below the damage threshold of the material.

• Typographical corrections: Please correct typographical errors throughout the text. For example: "Indeed, several recent experimental works have demonstrated light-induced spin switching in

CrI_3[27-29], showing that the material represents a practical experimental platform for investigating ultrafast spin dynamics."

This typo has been fixed ("the" replaced by "this"), and we have carefully searched the text for further typos.

• Line 73: "transitions from valence (v) to conduction (c)" — please use the same notation as in Fig. 2, namely valence band (VB) and conduction band (CB).

This typo has now been fixed.

• Line 199: "light" is misspelled as lihgth.

This typo has also now been fixed.

Reviewer #5 (Remarks to the Author):

In the present theoretical work, the Authors showed that the absorption of ultrashort laser pulses in the ferromagnetic semiconductors CrI_3 and CrSBr can lead to both an increase and a decrease in the magnetic moment, depending on the frequency of the laser. Special emphasis was placed on the increase in the moment, because this effect is as yet relatively unexplored. Two different approaches were chosen as methods: a tight-binding model and time dependent density functional theory, which both show similar dynamics of the magnetization and thus underpin the validity of the results. A central claim made by the authors is that they had identified a novel so-called “intraflip mechanism”, which can explain the laser-induced enhancement and decrease of the magnetic moment. In fact, the question about the physical mechanism is of fundamental interest. Therefore, I would generally assess that the submitted work could represent a significant advance in its field of research and that it would also be of considerable interest to a broad spectrum of physicists working on ultrafast dynamics. However, even in the resubmitted paper, I still see some significant unclarities in the interpretation and discussion of the mechanism. Therefore, I cannot recommend publication in Nature Communications unless the Authors have clarified these ambiguities. Before explaining the problematic points in detail, let me comment on the Authors' responses to the Referees' criticisms.

The responses to the first Reviewer's criticisms appear convincing. The Authors now explicitly mention that their calculations lead to similar results with both linearly and circularly polarized light. Because this shows that a direct transfer of angular momentum from light to the spin system is not essential, the physical conclusions on the mechanism become more precise. The other concerns of the first Reviewer have also been taken into account. For instance, calculations of the XMCD response function and more details of the laser pulse as well as of the material properties are now included in the revised version.

Concerning the fundamental objection raised by the second Reviewer, namely that too few materials were considered in this theoretical work, I do not share his opinion. On the contrary one should be aware that the Authors' main goal is not to highlight material-specific differences but to investigate and to understand the fundamental physics behind the effect. For this purpose, considering only two examples and a simple model, as presented by the Authors, seems to me to be entirely sufficient. Further comments of the second Reviewer were also addressed convincingly by the Authors.

However, in my opinion, the responses to the third Reviewer's comments were not always convincing. The first point raised by this Referee was that an electrical field applied to the fully occupied valence band cannot cause any change to the system unless an electron has been excited to the conduction band. Unfortunately, the Authors' response that the effect of Bloch acceleration on the electrons during the first half of the cycle and the effect during the second half of the cycle cancel each other out seems not applicable, as long as no electron-hole excitation has been generated, because there exists only a single many-electron state that describes the completely filled valence band (filled Fermi sea) which cannot change.

In any case, I share the Reviewer’s expectation that it is only through electron-hole excitation that a dynamic process is initiated, whereby only thereafter the hole in the valence band can change its wave vector and spin due to the Bloch acceleration effect.

This physical picture is supported by Figure 2(b) of the Supplemental Materials, which shows that the excited charge in the conduction band increases faster than the total spin moment. Importantly, according to this physical picture, the sequence of microscopic processes is exactly the opposite of that claimed by the Authors (who proposed that first the electron in the filled valence band changes

its spin and then, secondly, it is excited to the conduction band). Therefore, this point needs to be clarified. Either a truly convincing argument is required by the Authors or a reformulation of their interpretation.

We of course agree with the Referee that a transient electric field acting on a full band (i.e. the ground state of a semi-conductor/insulator) can change the system only in the event of electron-hole excitation, and not otherwise. As we describe below, the “intraflip” mechanism does not contradict this. Rather it relies on the fact that intraband motion accompanying electron-hole excitation can dramatically generated *change* the nature of electron-hole excitation.

To illustrate this we first provide a concrete example of this physics: the conversion of a electron-hole excitation that generates only charge excitation into one that generates both charge *and* current excitation [recently described by us in *Science Advances* **9** (11), eadf3673, (2023)], a so-called “hencomb” pulse. Subsequently, we discuss how the same physics underpins the “intraflip” mechanism that drives an ultrafast increase in moment in CrI_3 and CrSBr.

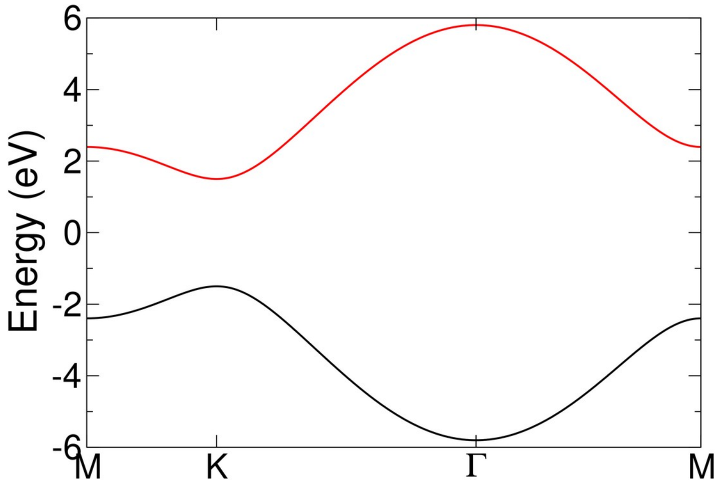

Fig. 1: Band structure of gapped graphene with the K point gap set to 3 eV.

Let us consider a minimal model of gapped graphene, a two band valley active system. The band structure of this system, with a gap of 3 eV, is shown in Fig. 1 above. Circularly polarized light excites charge at either the K or K^* valleys depending on the helicity of the pulse, and so this system represents the simplest model of the phenomena of light-valley coupling underpinning the field of valleytronics. Such light-wave excitations, however, cannot generate current: the excitation inherits the C_3 symmetry of the valley manifold and Bloch velocities cancel when integrated over momentum, leading to zero current after the pulse. This is shown in panels (a-c) of Fig. 2 below, where in panel (a) we show the laser pulse vector potential, in panel (b) the induced current, which is zero after the pulse, and in panel (c) the momentum resolved charge excitation.

If we consider a linear THz pulse of large amplitude, vector potential shown in panel (d), then this cannot excite charge across that gap as the THz frequencies (i.e. meV energies) of the pulse fall far below the 3 eV energy difference of valence and conduction bands at the valley. The electric field of the pulse is, however, large due to the large vector potential amplitude, $\mathbf{E}(t) = -\partial_t \mathbf{A}(t)$, with a peak electric field of 2.5 MV/cm. Nevertheless, as the Referee correctly states, this pulse produces no physical effect: the current is zero after the pulse and no charge is excited, panels (e) and (f) respectively. (Note we define here current as the expectation value of $\partial_{\mathbf{p}} H(t)$ for the time dependent wavefunction i.e. we do not include the $-\rho(t)\mathbf{A}(t)$ term.)

However, if we now at half cycle introduce the circularly polarized pulse described in panels (a-c), which yields an overall vector potential shown in panel (g), then the physics is dramatically different from either the circularly polarized pulse or the linear THz pulse: a large residual post-pulse current is now observed, panel (h), which occurs due to the shift off the valley centre of the charge excitation, panel (i). This post-pulse current arises as the cancellation of the Bloch velocities when integrated over the excited charge distribution can no longer occur due to the shift of the excitation off the valley centre. Thus by combining intraband motion (induced by the THz component) the the electron-hole excitation (induced by the circularly polarized component), the physical effect of the electron-hole excitation is dramatically altered.

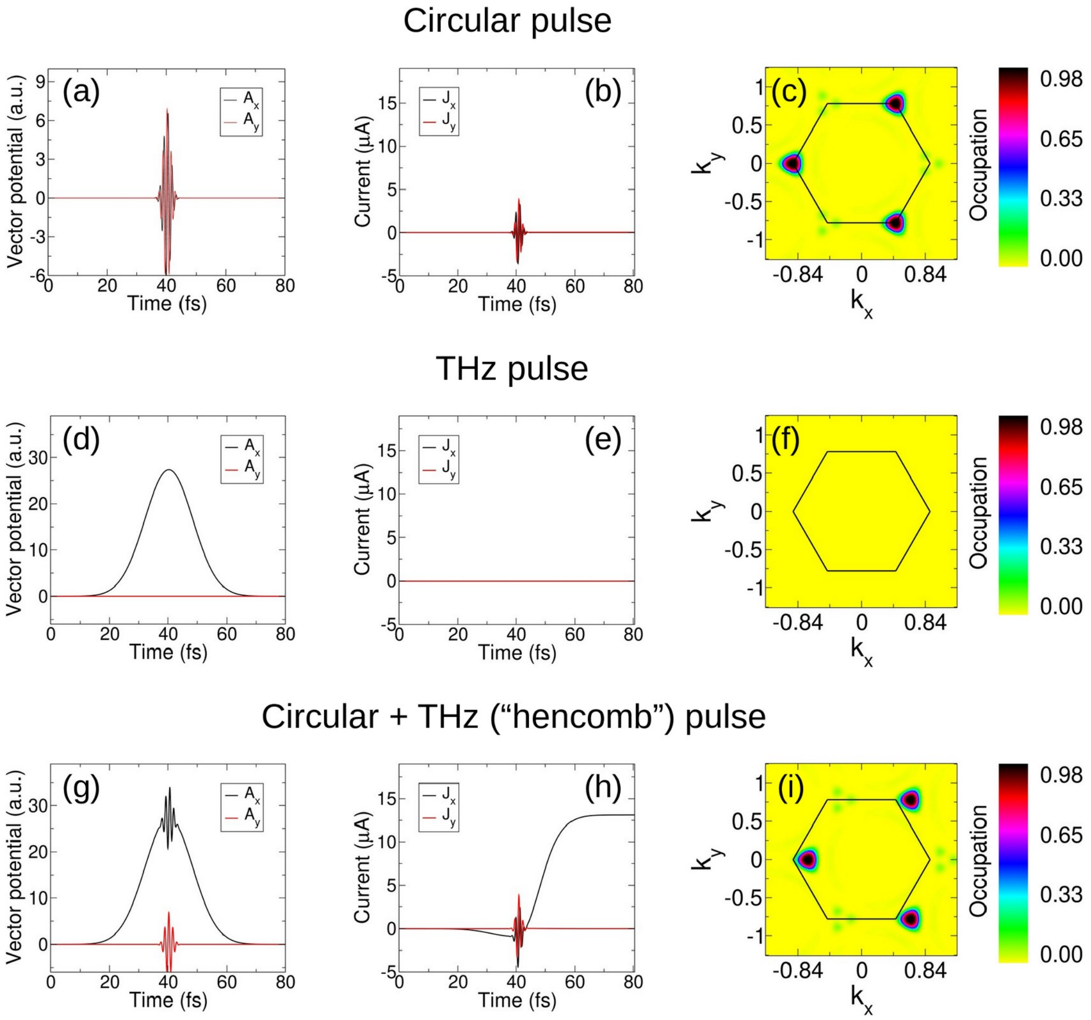

Fig. 2: *“Hencomb” excitation illustrating the role of a large electric field transient acting on a full band*. (a-c) A circularly polarized pulse applied to gapped graphene excites charge at one valley (in this case the K valley), but does not excite current as the C_3 symmetry of the charge excitation, inherited from the underlying valley manifold, results in complete cancellation of the microscopic current $\mathbf{j}_{\mathbf{k}}$when integrated over $\mathbf{k}$. (d-f) A Gaussian THz pulse can excite neither current or charge, as such a pulse acting on a full band generates no physical change. (g-i) The combination of these two pulses results in behaviour dramatically different from either acting alone. There now exists a strong post-pulse current, resulting from the fact that in momentum space the excited charge distribution has been shifted off the valley centre, breaking the symmetry cancellation of the circularly polarized pulse acting alone. This serves as an illustration of the fact that while a strong electric field transient generating intraband motion via the Bloch acceleration theorem acting alone cannot generate physical change, in combination electron-hole charge excitation it can result in dramatically different physics that the direct electron-hole charge excitation acting alone.

The origin of this effect can be understood by writing the time-dependent equation of motion in a Houston basis of Bloch functions as

$$i\partial_t \Psi(t) = H(\mathbf{k}(t))\Psi(t) \qquad \text{(Equation 2)}$$

where $\mathbf{k}(t) = \mathbf{k}(0) - \mathbf{A}(t)/c$. Evidently, the application of a pulse can result in two processes: (i) an evolution of the crystal momentum and (ii) electron-hole excitation. What occurs in the excitation shown in Fig. 2 represents a combination of these two processes which can be described in three steps, as illustrated schematically Fig. 3 below. There are: (1) the THz pulse evolves a k-vector $\mathbf{k}_0$, distant from the valley centre K, to the valley centre at half cycle. (2) The circularly polarized pulse, tuned to the gap at K, drives charge across the gap, after which (3) the second half cycle of the THz pulse then evolves the crystal momentum back to the original momentum $\mathbf{k}_0$. The net result is then a charge excitation centered on $\mathbf{k}_0$, exactly as seen in panels (g-i) of Fig. 2.

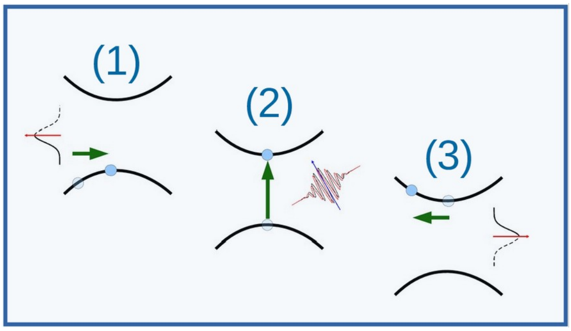

Fig. 3: Schematic of the "hencomb" excitation shown in Fig. 2. A pulse combining a THz component with, acting at half cycle a second gap tuned circularly polarized pulse, generates a sequence of (1) a half cycle of THz pulse driven intraband excitation, (2) a circularly polarized pulse interband excitation, and (3) a final half-cycle of the THz intraband excitation. Removing process (2), the interband excitation, results in a strong transient electric field applied to a full band and no physical change. However, the action of a circularly polarized at half cycle is dramatically different from when this acts alone, with the excitation shifted off the valley centre by the presence of the THz component.

This illustrates our central point that while a transient electric field by itself cannot change any physical property, in combination with a process that drives excitation across the gap i.e. electron-hole excitation, then physical properties are impacted by this transient electric field.

We now consider how this physics applies to CrI_3, and to this end employ a model that allows for the same two band analysis as described above. The band structure of CrI_3, at the simplest level, can be described as a valence sector featuring of spin up and spin down channel bands with significant spin hybridization, and a conduction sector consisting of pure spin up bands. An appropriate two band model therefore consists of one (the lower energy band) exhibiting spin hybridization between spin and and spin down, and a second pure spin band. We will then explore the dynamics of an initially occupied state in the lower band.

A two component vector potential is utilized, shown in panel (a), consisting of a sub-gap component (labeled "intraband") augmented by a second component at half cycle that excites charge (labeled "interband"), in short exactly the pulse design described above. The very different band structure, endowed with a spin texture, now brings dramatic new physics as compared to the spinless two band model of a valleytronic material.

The analysis of the action of this pulse can, just as in the case discussed above, can be described in three steps. First (1) the pulse drives an evolution of momentum from V_1 to V_2 which evolves the spin from down to up. (2) Electron-hole excitation, driven by the second pulse component, then excites charge into the conduction band, allowed as the spin has evolved from down to up. Subsequently (3) the second half cycle of the THz component then drives evolution of momentum

C_2 to C_1 in the conduction band with the conduction band charge remaining spin up as this band is spin pure, while in the valence band the remaining charge evolves from V_2 back to V_1 with evolution of the spin, in this band, from spin up back to spin down. This process is illustrated by the dashed line in Fig. 4 (a), which describes exactly the optically induced excitation of intraband evolution, interband excitation, and the intraband evolution described by steps (1-3). The overall result, however, is thus an optical excitation that results in the excitation of valence spin down charge at V_1 to a state in which some of the charge has been excited to spin up in the conduction band at C_2, indicated by the full arrow in Fig. 4 (a).

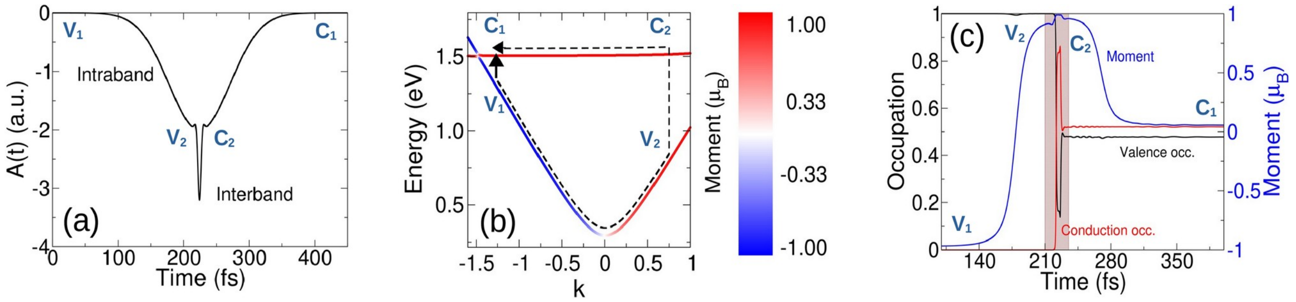

Fig. 4: *A model band structure exhibiting spin hybridization and the intraflip mechanism*. (a) A model laser pulse that generates an "intraflip" excitation consisting of two Gaussian envelopes. The weak electric field component, labeled "intraband", drives the required intra-band evolution of crystal momentum, but does not generate inter-band excitation. A second component possessing a strong electric field drives inter-band excitation from valence to conduction, labeled "interband". (b) Two possible light induced excitations within a spin hybridized electronic structure are indicated: a direct optical excitation from valence to conduction, $V_1 \to C_1$, forbidden as V_1 and C_1 have opposite spin. A second "intraflip" excitation is indicated by the dashed lines $V_1 \to V_2 \to C_2 \to C_1$. This excitation involves first an intra-band evolution from spin down to spin up ($V_1 \to V_2$) followed by an allowed spin up to spin up transition ($V_2 \to C_2$). After intra-band evolution $C_2 \to C_1$ this results in a spin flip excitation $V_1 \to C_1$, mediated by intra-band evolution of crystal momentum. (c) The change in occupation and moment during this "intraflip" process. Between V_1 and V_2 there is no change in occupation numbers -- the valence band occupation is 1 and the conduction 0 -- while the spin moment rotates from down to up. This is followed by excitation to the conduction band, indicated by the shaded area, in which the conduction occupation rises to ~ 0.5. Finally, in the second half cycle of the Gaussian pulse the moment reduces as the valence band state rotates back from up to down (along $V_2 \to V_1$) while the conduction band moment remains up ($C_2 \to C_1$). The overall effect is therefore a moment increase.

Tracking the temporal evolution of the charge excitation and magnetic moment of this process confirms exactly this picture, panel (c). The spin first evolves from down to up with no change in conduction band occupation, corresponding to the intraband evolution V_1 to V_2, with subsequently charge excitation occurring in the shaded area, corresponding to the interband excitation V_2-C_2 created by the second pulse component acting at half cycle of the THz component (the “spike” in the vector potential). There is then some reduction in moment as charge evolves back both valence band from V_2 to V_1 and from C_2 to C_1: the latter process does not change the spin but the former does, resulting in a reduction in moment from the value of 100% spin up found at half cycle.

One should note that by considering the dynamics of one particular k-vector and focusing on the dynamics of the topmost valence and lowermost conduction band, then we can clearly see the effect of the ultrafast light induced intra- and inter-band transitions. If, however, we considered all the valence bands and integrated over all k-vectors then the intraband motion occurring before electron-hole excitation will not produce any change in moment, exactly as the Referee suggests. However the presence of a electric field transient does change the response of the system at half-cycle, dramatically altering the physics of electron-hole excitations of the overall laser pulse, which this model, by focusing only on two-bands, highlights.

Thus the “intraflip” mechanism represents a second example of a strong electric field transient dramatically changing the physics of electron-hole excitation. In the first example, augmenting a circularly polarized pulse by a THz transient allowed a shift in momentum space of the charge excitation, endowing the excitation with (massive) current [*Science Advances* **9** (11), eadf3673, (2023)]. In the second example, the present paper, the effect of intra-band motion is even more dramatic: allowing excitation from spin down states into spin up states via an indirect optical path involving shifts in momentum space. We should stress that unlike the former case, the mechanism works when the same pulse component generates both the intra-band and inter-band excitation i.e. the linearly polarized pulse shown in Fig. 1 panel (b) of the manuscript. The complex spin texture of the valence bands in CrI_3 – see Fig. 1 panel (a) – requires only a pulse with large vector potential to generate intra-band motion, and a central frequency tuned to the band gap.

Following the suggestion also of Referee #2 we have included this discussion in the main body of the paper (Section 5, lines 187-215), and also added a new section to the supplemental in which we describe the example of the “hencomb” excitation discussed above (Section 9). We thank the Referee for focusing on the physics of intra-band motion and the role of the electric field acting on a full band and feel that by including this discussion in the manuscript the physics of the underlying mechanism is clarified.

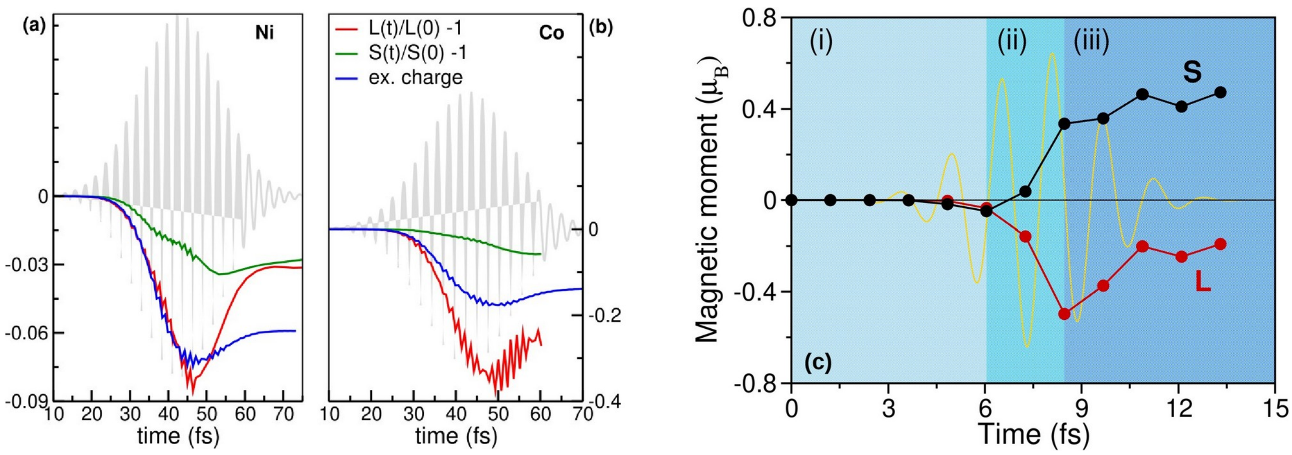

Fig. 5: *Early time spin and orbital angular momentum dynamics*. Shown are the temporal change in spin $\mathbf{S}$ and orbital angular momentum $\mathbf{L} = \mathbf{r} \times \mathbf{p}$, both evaluated within muffin-tin, for three materials Co and Ni – panels (a) and (b) respectively – and CrI_3, panel (c). In each case the laser pulse shown in background, and the calculations are performed using time-dependent density functional theory as implemented in the Elk code. For the elemental magnets Co and Ni the impact of the laser pulse is to generate a demagnetization, in both the orbital angular momentn and spin angular momentum. For CrI3 the dynamics of **L** and **S** are seen to be dramatically different, with three regimes of spin and angular momentum dynamics. (i) At early times both $\mathbf{L}$ and $\mathbf{S}$ exhibit a reduction, behaviour similar to that seen in demagnetization dynamics of elemental Ni and Co. (ii) This, however, is succeeded by a regime in which the spin angular momentum increases – generated by “intraflips” associated with the intraband evolution of momentum – while the orbital angular momentum decreases. The microscopic muffin-tin currents associated with this reduction in L would, via forces acting on the nuclei, transfer this reduction in angular momentum to the lattice, thus ensuring conservation of angular momentum. (Panels (a) and (b) are taken from Dewhurst *et al*. *Phys Rev B* **104**, 054438 (2021)).

The next issue raised by the third Reviewer concerns the question of which term in the Hamiltonian is responsible for the change in angular momentum during the enhancement of the total spin moment. Here, I agree with the Authors’ answer that the spin angular momentum is transferred to orbital angular momentum (by means of spin-orbit coupling), which then in turn is transferred to the lattice. However, their remark in the manuscript “the present TD-DFT calculations we do not include the dynamics of the lattice degrees of freedom, and thus the flow of spin angular momentum, via orbital degrees of freedom, to the lattice is not included in our calculations” seems a little too brief to me. It is understandable that consideration of nuclear degrees of freedom would go

beyond the scope of this work. However, it would certainly be helpful for the Reader if the Authors could discuss in more detail the different processes which might be relevant for that angular momentum flow from the spins to the lattice. The present explanation, that the changes in the spin moment take place basically during the Bloch acceleration of electrons, does not provide transparent insights into the involved microscopic processes.

While the clamping of the nuclei prohibits a complete discussion of the full physics of angular momentum, i.e. that of the nuclear angular momentum (**N**), as well as the orbital (**L**) and spin (**S**) angular momentum of the electron system, we can, as the Referee suggests, examine the dynamics of the spin (S) and orbital angular momentum separately, with a view to at least probing further into the physics underlying the changes in angular momentum.

We should stress at the outset that there are fundamental theoretical difficulties with regard to the analysis of angular momentum. As the position operator is undefined in a periodic solid (the operator $\mathbf{r}$ does not respect the crystal transition symmetry), then the operator for orbital angular momentum, $\mathbf{L} = \mathbf{r} \times \mathbf{p}$ is also not well defined in a periodic solid. This situation is resolved *at equilibrium* by the "modern theory of orbital magnetism" in which, either employing a Wannier basis or semi-classical approach, one has the expression[4]

$$\mathbf{L} = \sum_n \langle \boldsymbol{\nabla}_{\mathbf{k}} u_{n\mathbf{k}} | (H_{\mathbf{k}} + E_{n\mathbf{k}}) | \nabla_{\mathbf{k}} u_{n\mathbf{k}} \rangle \qquad \text{(Equation 3)}$$

where $|u_{n\mathbf{k}}\rangle$ is the periodic part of the Bloch function $|\psi_{n\mathbf{k}}\rangle = e^{i\mathbf{k}.\mathbf{r}}\,|u_{n\mathbf{k}}\rangle$ with $H_{\mathbf{k}} = e^{-i\mathbf{k}.\mathbf{r}} H e^{i\mathbf{k}.\mathbf{r}}$ and $H_{\mathbf{k}}\,|u_{n\mathbf{k}}\rangle = E_{n\mathbf{k}}\,|u_{n\mathbf{k}}\rangle$.

However, there is no corresponding expression for the non-equilibrium situation, and thus $\mathbf{L}(t)$ cannot at present be calculated for a periodic solid. This remains a fundamental outstanding theoretical challenge, as yet unresolved.

To bypass this we can, however, calculate a "poor mans" dynamical **L**, by calculating the temporal evolution of $\mathbf{L} = \mathbf{r} \times \mathbf{p}$ within the muffin-tin of Cr (i.e. a sphere placed around the atomic centre). We have previously employed this approach to study the angular momentum redistribution in the elemental magnets Ni and Co [Dewhurst *et al*. *Phys Rev B* **104**, 054438 (2021)], and have utilized this here, calculating $\mathbf{L}(t)$ and $\mathbf{S}(t)$ within the Cr muffin-tin of CrI_3, shown here as Fig. 5 panel (c) of this reply.

We first briefly discuss the corresponding case of the dynamics of **L** and **S** in the elemental magnets Ni and Co, panels (a) and (b) of Fig. 5. In the case of these simple magnets, both the spin and orbital angular momentum exhibit a laser induced demagnetization, with **L** seen to temporally "lead" **S**, exhibiting an enhanced demagnetization as compared to the former (note the y-axis shows the relative changes in moment). This arises as the laser pulse couples directly to the **L**, while only indirectly to the **S** via dynamical spin-orbit induced spin flips.

The case of CrI_3 exhibits dramatically different behaviour with, at first glance, light induced *magnetization* of **S** but *de-magnetization* of **L**. A closer examination reveals three distinct temporal domains, indicated by the background colour of the plot.

(i) At early times before the peak of the pulse spin and angular momentum both begin very slightly to decrease, behaviour that is reminiscent of the demagnetization dynamics of Ni and Co.

(ii) The "intraflip" mechanism then takes over, active at the peak of the pulse as this requires a large amplitude of the vector potential to derive intra-band evolution of crystal momentum, generating an increase in $\mathbf{S}(t)$. This increase in $\mathbf{S}(t)$ however is accompanied by a corresponding *reduction* in

orbital angular momentum $\mathbf{L}(t)$ (note the plot in Fig. 5 shows the change in these quantities). The dynamical excitation thus has generated microscopic currents within the muffin-tin that to some extent compensate for the increase in $\mathbf{S}(t)$. These microscopic currents generate forces on the nuclei, which would then, if the nuclei were not clamped, induce nuclear dynamic transferring angular momentum from the **L** of the electron system to the lattice. Thus ultimately the increase in **S** would be compensated for by a decrease in the angular momentum of the nuclear system.

(iii) Finally we see that **L**(t) begins to increase, as these forces have acted on the clamped nuclei, while the spin continues to increase somewhat.

This provides insight into the microscopic dynamical pathway by which the increase in spin angular momentum will ultimately be compensated by the lattice, and have therefore added Fig. (c) and this discussion as new section of the manuscript (Section 4, lines 162-186). We thank the Referee for encouraging us to provide a microscopic underpinning of the dynamics of **S**, **L**, and the lattice, and feel that by including this our paper may inspire further work on this outstanding problem.

In the following, I would like to make a few more comments in addition to the points already mentioned by the previous Reviewers.

i)
The intraflip mechanism has been presented by the Authors as if it were a novel mechanism. However, it is essentially based on a purely electronic Hamiltonian including the electronic bands and the spin-orbit interaction. These processes have not been considered for the first time but have already been used to explain conventional laser-induced demagnetization in metals. For this reason, I would consider it somewhat exaggerated to present the intraflip mechanism as a different mechanism. However, to my knowledge, its application to ferromagnetic semiconductors with their characteristic band structure is new and, therefore, worthy of publication.

While the mechanism is based, as the Referee says, on the electronic Hamiltonian including electronic bands and spin orbit interaction, a vast amount of the physics of early time dynamics (including physics presently undiscovered) will be based on such a Hamiltonian. A similar criticism could be aimed at, for instance, the OISTR effect (optical intersite spin transfer) which also arises out of exactly this Hamiltonian and generates ultrafast re-arrangements of magnetic structure. The same Hamiltonian thus generates two very different phenomena (moment increase and moment re-arrangement) which are accounted by for two strikingly different microscopic mechanisms ("intraflip" mechanism and OISTR).

This represents a rather general point: the same fundamental Hamiltonian can yield several distinct physical processes each described by their own microscopic mechanism. (Of course a rather clear limit of this would be the many body Hamiltonian of the electron system and lattice, which describes all of condensed matter physics). The ultimate point therefore is one of levels of theory, with a more fundamental Hamiltonian representing one level of theory, and distinct mechanisms of particular physical phenomena derived from this a second level of theory. A purely electronic Hamiltonian describing bands along with spin-orbit coupling represents the former case for a fundamental Hamiltonian; to see this consider the vast amount of physics arises from this Hamiltonian e.g. the spin-valley coupling underpinning valleytronics, Floquet states generated by periodic light pulses, topological insulators, and so on. To the best of our knowledge (i) a large moment increase induced by light has not previously been observed and (ii) the crucial role of intra-band transitions in this increase has not been described. We thus feel, and hope the Referee agrees, that the fact that the "intraflip" mechanism arises also from the fundamental does not ultimately detract from its novelty.

ii)
In the discussion the Authors claimed that the light pulse directly couples to the spin direction through the light pulse coupling to momentum, and that the intraflip effect occurs on a time scale dictated only by the pulse duration. These statements seem somewhat misleading, as they would imply that spin flips occur exclusively during the pulse. However, this is not always strictly true. For example, Figures 3(c), 3(g), and 4(g) show that the magnetic moment continues to change even after the pulse has ended. Of course, the change in the moment is then only slight, but not vanishing. From a theoretical perspective, the interaction of the electrons with the laser (which is independent of spin) and the spin-orbit interaction are actually independent of each other. In the present work, systems with a very strong spin-orbit coupling were considered. See, for example, beta = 0.45 eV in Table 1 of the Suppl. Materials. Could this be the reason why the magnetic moment decreases during the same time scale as the laser pulse acts, and therefore the intraflip effect seems to be induced by a direct coupling between electrons and the pulse? It would be clarifying for the Reader if the Authors would formulate more precisely their statement. Moreover, could the Authors share their expectations regarding how the results would be for systems with weaker spin-orbit coupling?

The reason for the moment varying after the pulse has ended results from interband terms in the time dependent expectation value of the moment. To see this we first note that this expectation value of the magnetic moment is

$$\mathbf{m}(t) = \sum_q \langle \Psi_\mathbf{q}(t) | \boldsymbol{\sigma} | \Psi_\mathbf{q}(t) \rangle \qquad \text{(Equation 4)}$$

where $|\Psi_\mathbf{q}\rangle$ is the time dependent wavefunction at a particular *initial* crystal momentum $\mathbf{q}$, and $\boldsymbol{\sigma}$ the vector of Pauli matrices $(\sigma_x, \sigma_y, \sigma_z)$. The time dependent wavefunction can be expressed in a Houston basis as

$$|\Psi_\mathbf{q}(t)\rangle = \sum_n c_{n\mathbf{q}}(t) \, |\phi_{n\mathbf{k}(t)}\rangle \qquad \text{(Equation 5)}$$

where $|\phi_{n\mathbf{k}(t)}\rangle$ are the local eigenstates at the time evolving k-vector $\mathbf{k}(t) = \mathbf{q} - \mathbf{A}/c$. These satisfy the ground state Hamiltonian $H_0 \, |\phi_{n\mathbf{k}}\rangle = \epsilon_{n\mathbf{k}} \, |\phi_{n\mathbf{k}}\rangle$. Inserting Eq. 5 into Eq. 4 then generates two terms

$$\begin{aligned} \mathbf{m}(t) &= \sum_\mathbf{q} \sum_n |c_{n\mathbf{q}}(t)|^2 \mathbf{m}_{n\mathbf{k}(t)} + \sum_\mathbf{q} \sum_{nn'} c^*_{n\mathbf{q}}(t) c_{n'\mathbf{q}}(t) \mathbf{m}_{nn'\mathbf{k}(t)} \\ &= \mathbf{m}_{intra} + \mathbf{m}_{inter} \end{aligned} \qquad \text{(Equation 6)}$$

where the first term is an intra-band term, summing only over a single band index and depending only on the occupation of the eigenstates $|c_{n\mathbf{q}}(t)|^2$, while the second term sums over two band indices n and n' and involves the product of expansion coefficients $c^*_{n\mathbf{q}}(t) c_{n'\mathbf{q}}(t)$. In each of these terms $\mathbf{m}_{n\mathbf{k}(t)} = \langle \phi_{n\mathbf{k}(t)} | \boldsymbol{\sigma} | \phi_{n\mathbf{k}(t)} \rangle$ and $\mathbf{m}_{nn'\mathbf{k}(t)} = \langle \phi_{n\mathbf{k}(t)} | \boldsymbol{\sigma} | \phi_{n'\mathbf{k}(t)} \rangle$ are matrix elements of the spin operator. After the pulse the occupation numbers no longer change, and therefore the first term is constant. However, the expansion coefficients are time dependent even after the pulse has ended due to the dynamical phase factors $e^{i\epsilon_{n\mathbf{q}} t/\hbar}$ of the basis eigenstates. The interband contribution to the total spin moment after the pulse can therefore be re-written with the time dependence explicitly shown as

$$\mathbf{m}_{inter} = \sum_\mathbf{q} \sum_{nn'} e^{-i(\eta_{n'\mathbf{q}} - \eta_{n\mathbf{q}})} e^{-i(\epsilon_{n'\mathbf{q}} - \epsilon_{n\mathbf{q}})t/\hbar} \mathbf{m}_{nn'\mathbf{k}(t)} \qquad \text{(Equation 7)}$$

Evidently this term retains a time dependence even after the pulse, driven by interband interference between the dynamical phases (the phases $\eta_{n'\mathbf{q}}$ and $\eta_{n\mathbf{q}}$ result from the excitation). This is the origin of the time dependence of the magnetic moment observed, for example, in Fig. 3 of the manuscript.

To demonstrate this numerically we can in the tight-binding method we calculate the intra- and inter-band contributions separately. To this end we take the results shown in Fig. 3 (g) of the manuscript, and present in Fig. 5 of the reply below the intra-band spin moment (dashed line) and the total spin moment (full line) as a function of time, panel (a), and the total excited charge, panel (b). The vector potential of the pulse is also shown (right hand axis) for temporal comparison with the excitation. As can be seen the intraband component does not change after the pulse, reflecting the fact that the intraflip mechanism that generates spin flip electron-hole excitation, i.e. excitation into the conduction band, occurs only during the pulse.

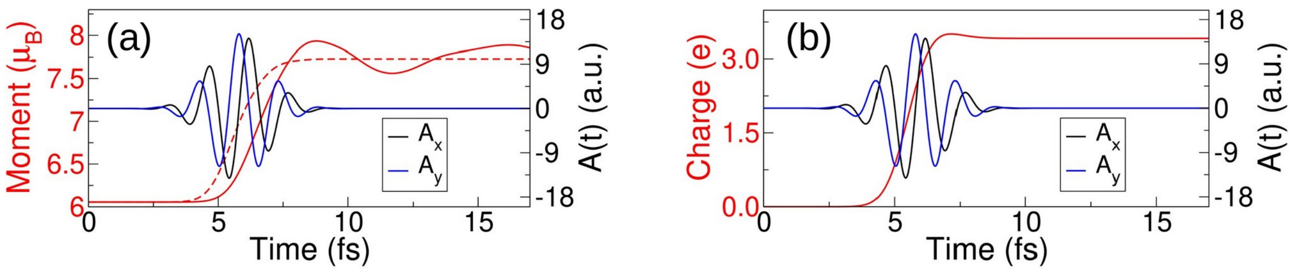

Fig. 6: (a) *Intra*band component of the magnetic moment, dashed line, along with the full magnetic moment (i.e. both *intra*- and *inter*-band components), full line. For temporal comparison the laser pulse that acts on the CrI_3 is shown also, referred to the right hand side axis. As can be seen the intra-band component, that depends on the occupancy of the band structure, remains unchanged after the pulse, with the full moment revealing oscillations about this value. These arise form interference between dynamical phases in the *inter*-band component of the moment, and do not reflect an excitation process. (b) The corresponding charge excitation which, just as for the intra-band component of the spin moment, is unchanged after the laser pulse.

The full moment, however, does change, and appears to oscillate about the intraband component of the magnetic moment, as one might expect from the structure of the intra- and inter-band terms in Eq. 4-5. These post-pulse changes, therefore, occur solely due to quantum interference arising from the creation of superposition excited state by the laser pulse. They are thus driven by the inherent dynamical phases associated with the energies of the states contributing to this exited state, and do not reflect an excitation process. They are therefore a feature only of the very early few femtosecond regime in which such coherent states exist, and quantum decoherence processes will damp out these oscillations about the intraband component at longer times.

This analysis of intra- and inter-band contributions to an early time physical observable is rather generic, and a very similar situation exists for the case of any operator, notably if we replace the spin operator $\boldsymbol{\sigma}$ by the current operator $\partial_p H$ the analysis into intra- and inter-band terms is one of the bedrock features of early time excitation of high harmonics[5].

We thank the Referee for drawing our attention to these post-pulse oscillations and we have now added a section to the Supplemental, Section 8, with appropriate reference in the main body of the paper, lines 126-130.

Finally, we address the question of the role of the strength of spin orbit coupling (SOC). A reduction in the SOC strength will act to reduce hybridization between spin-up and spin-down channels and, therefore, to reduce the mini-gaps that open at intersections of spin-up and spin-down bands.

Reducing SOC and the mini-gap will act to increase the spin-preserving transitions across the mini-gap leading, in turn, to a slowing down of the intraflip mechanism. To test this we have performed TD-DFT calculations of the spin dynamics with the SOC scaled by a pre-factor λ_{SO}. As can be seen in Fig. 7 of this reply, reducing the SOC strength in this way reveals the expected (slight) slowing down in the magnetic moment enhancement.

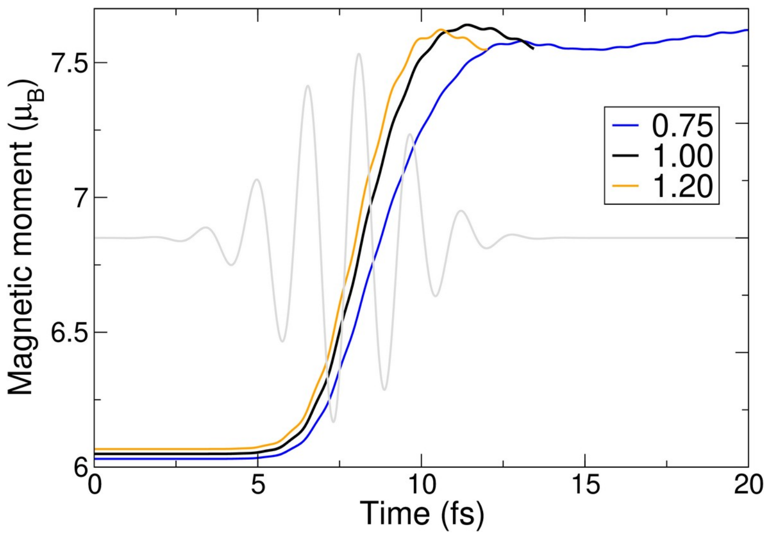

Fig. 7: (a) Moment enhancement for three values of a pre-factor multiplying the SOC term in the time dependent KS equation of TD-DFT: $\lambda_{SO} = 1.0$, which corresponds to the physical SOC of CrI_3, along with a case of suppression of the natural SOC, $\lambda_{SO} = 0.75$, and enhancement, $\lambda_{SO} = 1.20$. Suppression of SOC is seen to slow down slightly the enhancement, driven by the reduction in the spin-orbit induced mini-gaps that allows increased spin preserving transitions across the mini-gap, while, correspondingly, an increase in the strength of SOC acts to increase the mini-gaps and therefore make slightly faster the moment enhancement driven by the intraflip mechanism.

iii)
A minor comment: The two panels (b) and (c) of Figure 1 contain a full and a dotted curve, where the meaning of the dotted curves remains unclear. For the panel (c) it is written that the dotted curve illustrates the intra-band contribution to the magnetic moment. What is this? Does this refer to the contribution from the valence band? The Authors should explain their numerical results and discuss the physical implications.

This dashed curve is the intraband component of the magnetic moment, as now described in the response to the question about post-pulse changes in moment, see above. A full description of this has now been added to the Supplemental document, Section 8, and the caption amended to refer to this.

In conclusion, I recommend that the Authors clarify convincingly the above ambiguities. Without a corresponding revision, I cannot recommend publication in Nature Communications.

Reviewer #6 (Remarks to the Author):

I co-reviewed this manuscript with one of the reviewers who provided the listed reports. This is part of the Nature Communications initiative to facilitate training in peer review and to provide appropriate recognition for Early Career Researchers who co-review manuscripts.

Bibliography

(1) Barker, J.; Pashov, D.; Jackson, J. Electronic Structure and Finite Temperature Magnetism of Yttrium Iron Garnet. *Electron. Struct.* **2021**, *2* (4), 044002. https://doi.org/10.1088/2516-1075/abd097.

(2) Munawar, H. B.; Hussain, A.; Gouadria, S.; Noreen, S.; Bibi, N.; Tariq, A.; Tahir, M. B.; Rehman, J. U.; Arshad, S.; Ali, H. E. Structural, Electronic, Magnetic, and Optical Properties of $MF_{E_2}O_4$ (M = Ni, Fe, Co) Spinel Ferrites: A Density Functional Theory Study. *Int J of Quantum Chemistry* **2023**, *123* (14), e27124. https://doi.org/10.1002/qua.27124.

(3) Liu, C.; Gao, H.; Li, Y.; Wang, K.; Burton, L. A.; Ren, W. Manipulation of the Rashba Effect in Layered Tellurides MTe (M = Ge, Sn, Pb). *J. Mater. Chem. C* **2020**, *8* (15), 5143–5149. https://doi.org/10.1039/D0TC00003E.

(4) Ceresoli, D.; Thonhauser, T.; Vanderbilt, D.; Resta, R. Orbital Magnetization in Crystalline Solids: Multi-Band Insulators, Chern Insulators, and Metals. *Phys. Rev. B* **2006**, *74* (2), 024408. https://doi.org/10.1103/PhysRevB.74.024408.

(5) Yue, L.; Gaarde, M. B. Introduction to Theory of High-Harmonic Generation in Solids: Tutorial. *J. Opt. Soc. Am. B, JOSAB* **2022**, *39* (2), 535–555. https://doi.org/10.1364/JOSAB.448602.
